# Cassipourol and *β*-sitosterol from *Malva parviflora* L.: a mechanistic study of dual anti-inflammatory action against COX/LOX and TNF-*α*/BCL-2

**DOI:** 10.1038/s41598-026-56631-1

**Published:** 2026-06-13

**Authors:** Mohamed A. Anwar, Rania A. El Gedaily, Wael M. Aboulthana, Ahmed Elshewy, Zeinab A. Kandil, Shymaa I.A. Abdel-dayem

**Affiliations:** 1 Pharmacognosy Department, Faculty of Pharmacy, Cairo University, Kasr El Aini St., P.B. 11562, Cairo, Egypt; 2 Biochemistry Department, Biotechnology Research Institute, National Research Centre, 33 El Bohouth St., P.O. 12622, Dokki, Giza Egypt; 3 Pharmaceutical Organic Chemistry Department, Faculty of Pharmacy, Cairo University, Kasr El Aini St., P.B. 11562, Cairo, Egypt; 4 Natural and Applied Sciences Department, College of Arts and Sciences, The American University of Iraq-Baghdad (AUIB), Baghdad, Iraq

**Keywords:** *Malva parviflora*, Cassipourol, *β*-Sitosterol, Network pharmacology, Molecular docking, COX/LOX, Biochemistry, Cancer, Chemical biology, Chemistry, Computational biology and bioinformatics, Drug discovery

## Abstract

Although inflammation protects our bodies against harmful stimuli, uncontrolled inflammation drives serious chronic disorders. *Malva parviflora* L. (family Malvaceae) may represent a source for anti-inflammatory metabolites based on its potent anti-inflammatory activity. Its total ethanol extract demonstrated notable inhibition of cyclooxygenase-1/2 (COX-1/COX-2) and 5-lipoxygenase (5-LOX), with IC_50_ values comparable to those of the reference drugs indomethacin and zileuton, respectively. The hexane fraction was the most active fraction (lowest IC₅₀) among the solvent partitions (dichloromethane, ethyl acetate, and butanol). Subsequent column chromatography of the hexane fraction produced two compounds: Cassipourol, isolated for the first time from the Malvaceae family, and *β*-sitosterol. Their structures were confirmed by matching their NMR and mass spectrometry data with literature. Both were validated *in vitro* as dual COX/LOX inhibitors, exhibiting IC₅₀ values comparable to those of the standards. To uncover additional mechanisms, a compound–target–inflammation network was constructed using network pharmacology approaches, revealing 178 shared targets. Among these, tumor necrosis factor (TNF-*α*) and the antiapoptotic protein B-cell lymphoma 2 (BCL-2) emerged as central nodes linked to inflammatory pathways. Subsequent assays in human colon carcinoma (Caco-2) and lung adenocarcinoma (A549) cell lines showed that *β*-sitosterol suppressed TNF-*α* and BCL-2 by approximately 55%, whereas cassipourol displayed only modest inhibition (~20%). Molecular docking predicted moderate (*ca.* –4.5) and strong (> –5) binding affinities of both compounds to key inflammatory targets. Collectively, these results suggest that *β*-sitosterol from *M. parviflora* is a promising multitarget lead for inflammatory disorders, including cancer, whereas cassipourol requires further structural optimization and mechanistic investigation to improve its unfavorable physicochemical properties.

**Supplementary Information:**

The online version contains supplementary material available at 10.1038/s41598-026-56631-1.

## Introduction

Since ancient times, plants have represented a renewable source of active metabolites, especially secondary metabolites, which possess myriad pharmacological actions^1^. With advances in science, several studies focused on phytochemical investigations to isolate and identify these natural compounds and evaluate them as potential drug candidates using *in vitro*, *in vivo*, and/or *in silico* techniques^2–4^. Beyond their direct use in pharmaceutical products, these metabolites also inspire the synthesis of more potent derivatives^1^. This inspiration comes from structural complexity, chemical diversity, and varied biological activities of natural compounds^5–7^.

*Malva parviflora* L. (the Malvaceae family) is an annual herb native to North Africa, Europe, and temperate Asia, and is now naturalized in other temperate regions^8^. It has been incorporated into the Mediterranean diet, especially its leaves^9,10^. Several studies on *M. parviflora* reported the presence of various metabolites from different classes, including sterols, triterpenes, phenolic acids, flavonoids, and mucilage polysaccharides^11–17^. *M. parviflora* has demonstrated antioxidant and anti-inflammatory activities, showing efficacy against inflammatory disorders such as Alzheimer’s, diabetes, rheumatoid arthritis, ulcerative colitis, and wounds^12,16,18–21,^ which are in accordance with its traditional uses in treating inflammations, such as wounds and ulcers^22^.

Inflammation is a physiological protective response of the immune system to physical, chemical, and biological stimuli, such as stress, injury, and infection^23,24^. It is classified as either acute (persists for days) or chronic (persists for months or even years)^25^. Factors such as prolonged exposure to inflammatory stimuli or inappropriate immune response can trigger the transition of acute inflammation to the chronic stage^23,26^. This chronic inflammation contributes to numerous diseases, including atherosclerosis, autoimmune and neurodegenerative disorders, diabetes mellitus, and cancer^23,24,^ which account for over 50% of global deaths^27^.

Prostaglandin H_2_ synthases or cyclooxygenases (COXs) oxidize arachidonic acid into bioactive lipids called prostaglandins (PGs). Depending on the COX isoenzyme involved, the produced PGs can be protective or harmful. COX-1, a constitutive enzyme found in nearly all cell types, primarily produces housekeeping PGs essential for the stomach, kidney, and platelets. Additionally, it may produce other PGs that contribute to acute and neuroinflammation^28^. COX-2 is another isoform that is triggered by inflammatory stimuli. It generates pathological PGs that amplify inflammatory mediators, driving acute inflammations toward chronicity^29^. Thus, selective COX-2 inhibitors are considered safer due to fewer gastrointestinal side effects^30^. Moreover, 5-lipoxygenase (5-LOX) is another enzyme that metabolizes arachidonic acid into leukotrienes, key mediators of inflammatory disorders such as asthma and allergic rhinitis^31^.

Network pharmacology is an integrative *in silico* approach that integrates systematic medicine with information science^32^. Combining *in vivo* and *in vitro* experiments with computational analysis establishes a comprehensive network between drug candidates and diseases with common protein targets. Analyzing these networks helps identify potential targets and molecular mechanisms of the studied compounds^33,34^. Molecular docking is another computational tool used to predict the conformation and binding affinity of ligands (drugs) and protein targets^35^, revealing the biochemical and molecular mechanisms underlying their binding^36,37^.

Building on the established medicinal potential of *M. parviflora*, this study investigates the chemical composition and pharmacological properties of the Egyptian species to identify the compounds responsible for its anti-inflammatory activity and evaluate them as potential therapeutic candidates. Our research integrates phytochemical isolation, *in vitro* enzymatic and cellular validation, network pharmacology, and molecular docking to comprehensively investigate the anti-inflammatory potential of the isolated compounds. We began by screening various extracts *in vitro* against the “gatekeeper” inflammatory enzymes: COX-1, COX-2, and 5-LOX. The hexane fraction proved the most potent activity, enabling the targeted isolation of its major secondary metabolites. However, recognizing that plant-based remedies rarely act through a single target, we adopted a network pharmacology approach to explore how these compounds might interact with a broader array of human proteins. This approach highlighted TNF-*α* and BCL-2 as central molecular targets. Such findings allowed us to logically translate our study from simple enzyme inhibition to more complex cell-signaling and survival pathways. By combining molecular docking with targeted *in vitro* validation, we provided a more comprehensive picture of the multitarget anti-inflammatory mechanism of *M. parviflora*.

## Materials and methods

### Chemicals of the phytochemical study

All solvents used in this study, i.e., ethanol, methanol, hexane (60–80 °C), methylene chloride (dichloromethane), ethyl acetate, and *n*-butanol, were of pure analytical grade and were purchased from Piochem (6th of October City, Giza, Egypt). Precoated silica gel 60 GF254 (20 × 20 cm, 0.25 mm thickness) for thin layer chromatography (TLC), silica gel 60 H for vacuum liquid chromatography (VLC), and silica gel 60 (0.063–0.200 mm) for column chromatography were supplied by E. Merck (Darmstadt, Germany).

### Plant material

Fresh leaves of *M. parviflora* L. were collected in January 2023 from a farm in Giza governorate, Egypt. Prof. Dr. Wafaa M. Amer, Professor of Plant Taxonomy and Flora, Faculty of Science, Cairo University, kindly authenticated the plant. A voucher specimen was deposited in the Herbarium of the Pharmacognosy Department, Faculty of Pharmacy, Cairo University, Egypt (registration number: 1.6.23). The leaves were washed and air-dried before being ground into a fine powder.

### Extraction of *M. parviflora* and fractionation of its extract

As shown in (Suppl. Fig. S1), the leaf powder (1 kg) was repeatedly extracted till exhaustion by cold maceration in 70% ethanol (5 × 3 L). The combined extract was filtered and concentrated using a rotary evaporator (SCILOGEX RE100-Pro, Washington, USA) at a temperature not exceeding 60 °C, yielding 80 g of dry extract. A total of 70 g of the dry extract was suspended in a mixture of 300 mL distilled water and 30 mL methanol and fractionated with various organic solvents. The suspension was successively shaken with hexane (60–80 °C, 8 × 500 mL), methylene chloride (4 × 250 mL), ethyl acetate (3 × 250 mL), and saturated *n*-butanol (4 × 250 mL). Then, the resulting fractions were concentrated using the rotary evaporator at a temperature not exceeding 40 °C to produce 30 g, 2 g, 1 g, and 9 g, respectively.

Based on extract yield and *in vitro* anti-inflammatory activity, the hexane fraction was selected for further chromatographic isolation of its bioactive constituents. A 25 g portion was subjected to vacuum liquid chromatography (VLC) using a silica gel 60 H column (7.5 × 20 cm, 125 g). Elution was performed with a gradient system starting from 100% hexane, followed by stepwise increases in polarity using methylene chloride (10% increments) up to 100%. Then, ethyl acetate was added to methylene chloride in similar increments up to 100%, with 100 mL collected at each step, yielding 21 subfractions.

The separation was monitored by TLC using hexane/ethyl acetate (8:2, *v/v*). Subfractions 12–14 afforded 50 mg of cassipourol, while subfraction 16 yielded 57 mg of *β*-sitosterol, resulting in the isolation of two major compounds.

Subfractions 12–14 (eluted with 10–30% ethyl acetate in methylene chloride) were combined and subjected to a second VLC step. A 1.5 g portion was chromatographed on a smaller silica gel 60 H column (3.5 × 20 cm, 30 g) using a gradient system starting with 100% hexane and gradually increasing ethyl acetate to 10% (100 mL per step), yielding 23 subfractions. TLC monitoring (hexane/ethyl acetate 8:2, *v/v*) identified Subfraction 4 (1.5% ethyl acetate in hexane) as containing a major violet spot (Rf 0.6) after derivatization with *p*-anisaldehyde/H₂SO₄ and heating at 100 °C. This fraction (240 mg) was further purified on a silica gel 60 column (25 mL burette, 5 g) using 1% ethyl acetate in hexane, yielding Compound 1 (50 mg).

Similarly, 1.3 g of fraction 16 (eluted with 50% ethyl acetate in methylene chloride) was subjected to VLC under the same conditions used for Subfractions 12–14. Based on TLC analysis, Subfraction 8 (150 mg, eluted with 7% ethyl acetate in hexane) was selected for further purification on a silica gel column (2.5 × 20 cm, 25 g). Elution with 3% ethyl acetate in hexane afforded Compound 2 (57 mg). TLC (hexane/ethyl acetate 8:2, *v/v*) showed a violet spot (Rf 0.44) after visualization with *p*-anisaldehyde/H₂SO₄ and heating.

### NMR analysis

Nuclear magnetic resonance (NMR) spectra were recorded on a Bruker Ascend NMR spectrometer (U28-E04, ^1^H 400 MHz and ^13^C 100 MHz) using deuterated chloroform (CDCl_3_, Sigma-Aldrich, St. Louis, USA) as the solvent.

### COX-1, COX-2, and 5-LOX enzyme assays

The *in vitro* anti-inflammatory activity of the ethanol extract, its fractions (hexane, methylene chloride, ethyl acetate, and *n*-butanol), and the isolated compounds (cassipourol and *β*-sitosterol) were evaluated by measuring their inhibitory effects on COX-1 (ovine), COX-2 (human), and 5-LOX (human recombinant) enzymes. Inhibition percentages were calculated according to manufacturers’ instructions. Commercial assay kits were used for enzyme evaluation: COX-1/COX-2 (catalog number 560101, Cayman Chemical, Ann Arbor, MI, USA) and 5-LOX (catalog number 437996, Sigma-Aldrich)^38^. Briefly, for COX assays, the reaction mixture consisted of 10 µL enzyme (COX-1 or COX-2) and 960 µL of 0.1 M buffer (pH 8.0). Subsequently, 10 µL of each tested sample (100 µg/mL) was added, followed by incubation at room temperature for 10 min. The reaction was initiated by adding 10 µL arachidonic acid (0.1 M), followed by 50 µL HCl (1 M) and 50 µL Ellman’s reagent. Absorbance was measured at 410 nm against a blank using a spectrophotometer (Shimadzu UV–Vis, UV-240).

For the 5-LOX assay, 90 µL enzyme solution was mixed with 100 µL de chromogen, followed by addition of the tested sample (100 µg/mL). The reaction was initiated by adding 10 µL arachidonic acid (0.1 M) and incubated with gentle shaking for 10 min. Absorbance was recorded at 490 nm against a blank.

IC₅₀ values were determined by testing a range of concentrations (5–50 µg/mL). Dose–response curves were analyzed using linear regression. Indometacin and zileuton (Sigma-Aldrich, St. Louis, USA) was used as a reference drug for COXs and 5-LOX assays, respectively. All measurements were performed in triplicate, and each assay was repeated at least three times in independent experiments. Data is expressed as mean ± standard error (SE).

### Network pharmacology

Construction of this network was guided by the study of Khalil et al.^34^, using reported databases and tools in the review of Zhao et al.^33^.

#### Identification of inflammation-associated targets

To identify inflammation-associated targets, “Inflammation”^39^ was used as a search term in the search box of the GeneCards (https://www.genecards.org/) and DisGeNET (https://disgenet.com/) databases (both accessed on November 23, 2024). The resulting inflammation-related targets were downloaded, merged, and dereplicated, yielding a total of 10,213 targets.

#### Identification of potential targets for the isolated compounds

To identify the targets of cassipourol and *β*-sitosterol, their SMILES notations were obtained from PubChem (https://pubchem.ncbi.nlm.nih.gov/). These notations were imported into the Swiss Target Prediction (http://www.swisstargetprediction.ch/) and Herbal Ingredients’ Targets (HIT 2.0) (http://hit2.badd-cao.net/) databases (both accessed on November 23, 2024), and the resulting potential protein targets were downloaded, merged, and dereplicated to produce a total of 185 targets.

#### Construction of compounds-targets-inflammation and PPI networks

To determine the common targets between the two isolated compounds and inflammation, the two lists resulting from the two previous Sections were uploaded to the Venny 2.1 platform (https://bioinfogp.cnb.csic.es/tools/venny/, accessed on November 23, 2024) to construct a Venn diagram. Next, the targets of each compound were filtered to match the resulting common inflammation targets. Finally, these filtered compound-target lists and the common inflammation targets were imported into Cytoscape 3.10.3 to create the Two Compounds–Targets–Inflammation networks and further analyze them.

Regarding the protein-protein interaction (PPI) network, the common inflammation targets list was uploaded to the STRING database (https://string-db.org/, accessed on November 23, 2024). The biological species was selected as “*Homo sapiens*” and the confidence limit was set to ≥ 0.7. The produced network was exported to Cytoscape software for visualization and analysis.

#### Enrichment of GO terms and KEGG pathways

To perform Gene Ontology (GO) and Kyoto Encyclopedia of Genes and Genome (KEGG) enrichment analyses, the common inflammation targets list, generated in the previous Section, was uploaded to the ShinyGO 0.81 online tool (http://bioinformatics.sdstate.edu/go/, accessed on November 23, 2024) using the default parameters.

### *In vitro* activity of isolated compounds against TNF-*α* and BCL-2

To assess inhibitory activity against inflammation-related targets, tumor necrosis factor-*α* (TNF-*α*) and B cell lymphoma-2 antiapoptotic protein (BCL-2), human colon carcinoma (Caco-2) and lung adenocarcinoma (A549) cell lines (ATCC, Manassas, VA, USA) were used. Cells were cultured in Dulbecco’s Modified Eagle’s Medium (DMEM medium) supplemented with 25 mM glucose, 10% fetal bovine serum (Gibco, Grand Island, NY, USA), and 1% penicillin and streptomycin (Sigma, Deisenhofen, Germany). Cells were maintained at 37 °C in a humidified atmosphere containing 5% CO₂. Cells between passages 20 and 25 were used for all experiments.

IC_50_ values for the cytotoxic activity of the isolated compounds and indomethacin (standard anti-inflammatory drug) were evaluated using the 3-(4,5-dimethylthiazol-2-yl)-2,5-diphenyltetrazolium bromide (MTT) assay as previously described^40^. Cells were seeded in 96-well plates at a density of 3 × 10⁴ cells/well and incubated with 10 µL of various concentrations of each compound in serum-free medium for 48 h at 37 °C. After incubation, the medium was removed, and 40 µL of MTT solution (2.5 mg/mL) (Sigma-Aldrich, St. Louis, USA) was added to each well, followed by a 4 h incubation. The resulting formazan crystals were dissolved in 200 µL dimethyl sulfoxide (DMSO; cat# D8418, Sigma-Aldrich IT), and absorbance was measured at 570 nm using a microplate reader (SpectraMax Paradigm Multi-Mode).

Cell viability (%) was calculated as follows:

$$\mathrm{Viability}(\%) = (\mathrm{OD}_{\mathrm{sample}}/\mathrm{OD}_{\text{control (no drug))}} \times 100.$$

IC₅₀ values were determined by plotting cell viability against concentration (Suppl. Tables S3 and S4).

At their respective IC₅₀ values, the effects of the tested compounds on levels of TNF-*α* (Catalog **#** KHC3011) and BCL-2 (Catalog **#** BMS244-3) were evaluated using ELISA kits (both are human type, from Invitrogen Corporation), following the manufacturer’s instructions. DMSO was used as a negative control. All experiments were performed in triplicate, and results are expressed as mean ± SE.

### Molecular docking

Molecular docking studies were conducted using Molecular Orbital Environment (MOE) software v. 2014.0901 (Chemical Computing Group, Montreal, Canada). Consistent with the *in vitro* studies, indomethacin and zileuton were employed as clinically approved reference anti-inflammatory drugs against COXs and 5-LOX, respectively^41,42^. For TNF-*α* and BCL-2, indomethacin was used as a comparative small-molecule benchmark^43^. The 2D structures of cassipourol, *β*-sitosterol, indomethacin, and zileuton were downloaded from PubChem (https://pubchem.ncbi.nlm.nih.gov/) as SDF files. For docking preparation, the energy of each molecule was minimized using the MMFF94x force field until a root-mean-square deviation (RMSD) gradient of 0.05 kcal·mol 1Å 2 was reached, and the partial charges were calculated automatically^44^. Human crystal structures of COX-1 (ID: 6Y3C)^45^, COX-2 (ID: 5F19)^46^, 5-LOX (ID: 6N2W)^47^, TNF-*α* (ID: 2AZ5)^48^, and BCL-2 (ID: 6GL8)^49^ were retrieved from the Protein Data Bank (https://www.rcsb.org) as PDB files. Each protein was initially prepared by removing unincluded H_2_O molecules and ligands, then by using the QuikPrep protocol in MOE with the default options^44,50^. Docking was performed using ligand-bound (holo) protein structures for COX-2, 5-LOX, TNF-*α*, and BCL-2. The active sites were defined based on the retained cocrystallized ligands. Since COX-1 lacked a cocrystallized ligand, its active site was defined using the Site Finder tool with default parameters^44,51^. The Triangle Matcher placement method and London dG scoring function were used for docking^44^. The most stable docking poses were selected based on the conformation with the best score calculated by the MOE scoring function. To validify the protocol, redocking of the cocrystallized or reference ligands was performed and the RMSD values were 1.7736, 0.9456, 1.7663, 3.9418, and 2.8675 for COX-1, COX-2, 5-LOX, TNF*-α*, and BCL-2, respectively.

### Statistical analysis

Significant differences between groups were assessed using one-way analysis of variance (ANOVA) followed by Tukey’s post hoc test to compare significance among different treatment groups. A *p*-value of less than 0.05 was considered to indicate statistical significance.

## Results and discussion

### Identification of isolated compounds

Phytochemical investigation led to the isolation of two compounds. Compound 1 was isolated as a colorless, viscous liquid. Its structure was determined using a combination of ^1^H, ^13^C, heteronuclear single quantum coherence (HSQC), homonuclear correlation spectroscopy (COSY), and heteronuclear multiple bond correlation (HMBC) NMR. The analysis of these data was consistent with previously published data for cassipourol^52^. The ^1^H NMR spectrum (Suppl. Fig. S2) revealed an olefinic proton at *δ*_*H*_ 5.4 (t, *J* = 7.0, 7.0 Hz) and an oxy methylene group at *δ*_H_ 4.1 (d, *J* = 7.0). The methyl region included three singlets (*δ*_H_ 0.87, 0.88, and 1.66) and two doublets (*δ*_H_ 0.85, *J* = 6.5 Hz; 0.87, *J* = 1.7 Hz). The ^13^C spectrum (Suppl. Fig. S3) displayed signals for the double bond carbons at *δ*_c_ 140.21 (quaternary) and 123.04 (tertiary), and the carbon of the oxy methylene group at *δ*_c_ 59.34. The HSQC spectrum (Suppl. Fig. S4) confirmed the expected direct correlations between these key carbons and their corresponding hydrogens. The remaining protons and carbons were assigned with the aid of the COSY (Suppl. Fig. S5) and HMBC (Suppl. Fig. S6) spectra (assignments displayed in Table 1).

**Table 1:** ^1^H,^13^C NMR data for compounds 1 and 2 (CDCl_3_, ^1^H 400 MHz, ^13^C 100 MHz, *δ* in ppm and *J* in Hz).

Position		Cassipourol (1)				β-Sitosterol (2)		
	Type	δ ^1^H	Ref. ^52^	δ ^13^C	Ref. ^52^	Type	δ ^1^H	Ref. ^14^
1	QC	–	–	36.78	36.74	CH_2_	1.46 (m)	1.46
2	CH_2_	1.15 (m)	1.28	39.36	39.45	CH_2_	1.56 (m)	1.56
3	CH_2_	1.29 (m)	1.26	24.78	24.87	CH(OH)	3.54 (m)	3.54
4	CH_2_	1.09 (m)	1.24	37.42	37.44	CH_2_	2.33 (m)	2.32
5	CH	1.55 (m)	1.50	27.96	28.06	QC (=)	–	–
6	CH	1.39 (m)	1.38	32.78	32.88	CH (=)	5.37 (d, 5)	5.37
7	CH_2_	1.29 (m)	1.28	24.45	24.55	CH_2_	2.04 (m)	2.04
8	CH_2_	1.39 (m)	1.34	37.36	37.44	CH	1.66 (m)	1.69
9	CH	1.39 (m)	1.40	32.68	32.78	CH	1.56 (m)	1.55
10	CH_2_	1.39 (m)	1.38	37.42	37.51	QC	–	–
11	CH_2_	1.5 (m)	1.53	25.14	25.21	CH_2_	1.51 (m)	1.52
12	CH_2_	2 (m)	1.99	39.87	39.95	CH_2_	1.51 (m)	1.51
13	QC (=)	–	–	140.21	140.42	QC	–	–
14	CH (=)	5.41 (t, *J* = 7.0, 7.0)	5.40	123.04	123.16	CH	1.27 (m)	1.50
15	CH_2_	4.15 (d, *J* = 7.0)	4.14	59.34	59.522	CH_2_	1.27 (m)	1.58
16	CH_3_	0.88^a^ (s)	0.86	22.61^a^	22.80	CH_2_	1.85 (m)	1.85
17	CH_3_	0.87^a^ (s)	0.85	22.70 ^a^	22.70	CH	1.27 (m)	1.45
18	CH_3_	0.87^b^ (d, 1.7)	0.84	19.62 ^b^	19.83	CH_3_	0.70 (s)	0.70
19	CH_3_	0.85^b^ (d, *J* = 6.5)	0.83	19.73 ^b^	19.80	CH_3_	1.03 (s)	1.03
20	CH_3_	1.67 (br, s)	1.66	16.15	16.26	CH	1.66 (m)	1.60
21						CH_3_	0.94 (d, 6.5)	0.94
22						CH_2_	0.93 (m)	0.93
23						CH_2_	1.15 (m)	1.15
24						CH	1.15 (m)	1.38
25						CH	1.6 (m)	1.57
26						CH_3_	0.84 (d, 1.5)	0.84
27						CH_3_	0.86 (overlapping d)	0.86
28						CH_2_	1.05 (m)	1.10
29						CH_3_	0.94 (overlapping t)	0.82

Assignments were made based on HSQC, COSY and HMBC spectroscopic data in comparison with the literature. Value with the same superscript letter may be interchangeable.

**Fig. 1:**
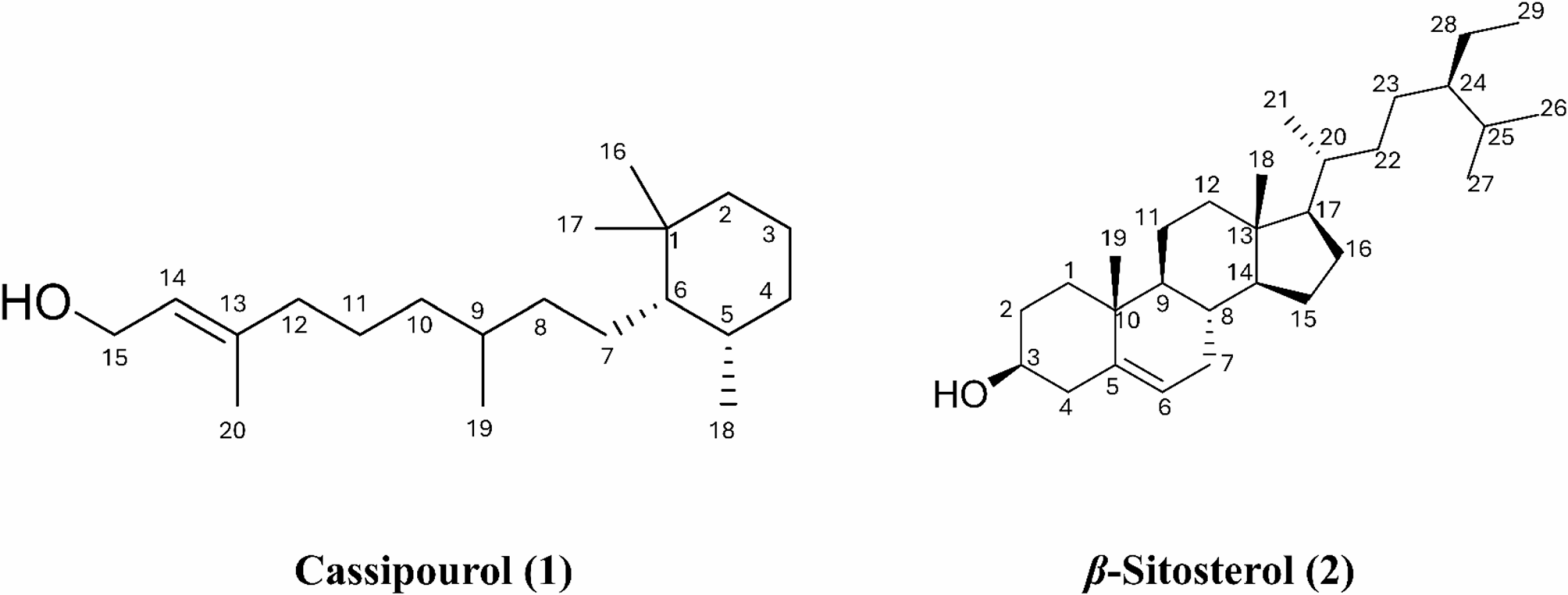
The chemical structures of compounds 1 & 2.

Key COSY correlations were observed: H-3/H-4; H-5/H-6; H-5/H-6 with H-18; H-9/H-19; H-11/H-12; and H-14/H-15. Significant C-H cross peaks in the HMBC spectrum included correlations from H-2 to C-1 and C-16/C-17; H-3 with C-4; H-4 with C-2, C-6, and C-16/C-17; H-5 with C-16/C-17; H-6 and H-7 with C-5; H-10 with C-12; H-11 with C-12 and C-20; H-12 with C-10, C-11, C-13, C-14, and C-20; H-14 with C-12 and C-20; H-15 with C-13 and C-14; H-16/H-17 with C-1, C-2 and C-6; H-18 with C-4, C-5, and C-6; H-19 with C-8 and C-9; H-20 with C-13, C-12, and C-14, confirming the monocyclic structure. Compound 1 was identified as cassipourol (Fig. 1), a monocyclic diterpene, with the molecular formula C_20_H_38_O. This identification was further confirmed by electron ionization mass spectrometry (EI-MS), which revealed (M^+^) *m/z* 293.99, corresponding to cassipourol (Suppl. Fig. S7). To the best of our knowledge, this study is the first to report the presence of monocyclic diterpene, cassipourol, within the family Malvaceae.

Compound 2 was obtained as colorless needle crystals and identified as *β*-sitosterol (Fig. 1). This identification was primarily based on matching its ^1^H NMR data (Suppl. Fig. S8) with a previously published study of *β*-sitosterol isolated from *M. parviflora* root bark^14.^ The ^1^H NMR spectrum revealed signals for six methyl groups, eleven methylenes, and nine methines (Table 1). Characteristic features included singlets at *δ*_*H*_ 0.70 and 1.03, corresponding to two methyl groups attached to quaternary carbons. A multiplet at *δ*_*H*_ 3.54 is due to a proton connected to a hydroxylated carbon. An overlapping triplet signal at *δ*_*H*_ 5.37 indicates an olefinic proton. The compound identity was further confirmed by TLC spotting against an available reference standard. Additionally, EI-MS provided a molecular ion peak (M^+^) 413.99 *m/z*, consistent with the molecular weight of *β*-sitosterol (Suppl. Fig. S9).

### *In vitro* COX-1, COX-2, and 5-LOX enzyme assays of ethanol extract and its fractions

Table 2 presents the *in vitro* anti-inflammatory activity of the ethanol extract of *M. parviflora* and its fractions against COX-1, COX-2, and 5-LOX enzymes. The total ethanol extract exhibited the strongest overall activity among all tested samples. It inhibited COX-1 (60.73% at 100 µg/mL; IC₅₀ = 6.63 µg/mL) and COX-2 (62.98%; IC₅₀ = 4.93 µg/mL), with values closely comparable to indomethacin (COX-1: 61.16%, IC₅₀ = 6.58 µg/mL; COX-2: 63.41%, IC₅₀ = 4.90 µg/mL). Its 5-LOX inhibition (55.83%; IC₅₀ = 7.17 µg/mL) was also comparable to zileuton (48.26%; IC₅₀ = 8.30 µg/mL), indicating dual COX/LOX inhibitory potential. Among the fractions, the hexane fraction exhibited moderate activity, with inhibition of COX-1 (48.48%; IC₅₀ = 8.30 µg/mL), COX-2 (50.73%; IC₅₀ = 6.12 µg/mL), and 5-LOX (43.58%; IC₅₀ = 9.19 µg/mL). In contrast, the methylene chloride, ethyl acetate, and *n*-butanol fractions showed comparatively weak activity, with higher IC₅₀ values.

**Table 2:** *In vitro* COX-1, COX-2 and 5- LOX enzyme assays of *M. parviflora* leaf ethanol extract and its fractions.

	Anti-inflammatory activity					
	COX-1		COX-2		5-LOX	
	Inhib. (%)	IC_50_ (µg/mL)	Inhib. (%)	IC_50_ (µg/mL)	Inhib. (%)	IC_50_ (µg/mL)
Total ethanol extract	60.73 ± 0.57	6.63 ± 0.08	62.98 ± 0.57	4.93 ± 0.07	55.83 ± 0.57	7.17 ± 0.11
Hexane fraction	48.48 ± 0.46^ab^	8.30 ± 0.10^ab^	50.73 ± 0.46^ab^	6.12 ± 0.09^ab^	43.58 ± 0.46^ab^	9.19 ± 0.14^ab^
Methylene chloride (Dichloromethane)	27.97 ± 0.27^abc^	14.38 ± 0.17^abc^	30.22 ± 0.27^abc^	10.28 ± 0.15^abc^	23.07 ± 0.27^abc^	17.36 ± 0.28^abc^
Ethyl acetate	13.24 ± 0.13^abcd^	30.39 ± 0.37^abcd^	15.49 ± 0.13^abcd^	20.06 ± 0.28^abcd^	8.34 ± 0.13^abcd^	48.03 ± 0.97^abcd^
Butanol	16.68 ± 0.16^abcde^	24.13 ± 0.29^abcde^	18.93 ± 0.16^abcde^	16.41 ± 0.23^abcde^	11.78 ± 0.16^abcde^	34.00 ± 0.63^abcde^
Indomethacin	61.16 ± 0.10	6.58 ± 0.02	63.41 ± 0.10	4.90 ± 0.03	–	–
Zileuton	–	–	–	–	48.26 ± 0.10	8.30 ± 0.04

Values are expressed as mean ± standard error (SE) from three independent replicates. Indomethacin and zileuton were used as reference drugs for COX-1/2 and 5-LOX assays, respectively. Percentage inhibition (Inhib. %) was calculated at a fixed concentration of 100 µg/mL. Statistical significance is indicated as follows: ^a^, significantly different from the standard drug; ^b^, significantly different from the ethanol extract; ^c^, significantly different from the hexane fraction; ^d^, significantly different from the methylene chloride fraction; ^e^, significantly different from the ethyl acetate fraction (*p* ≤ 0.05).

Collectively, the total ethanol extract and hexane fraction showed the most promising anti-inflammatory activity, possibly due to synergistic effects of bioactive constituents. Based on these results, the hexane fraction was selected for further isolation and characterization of its active metabolites.

Previous studies demonstrated the anti-inflammatory potential of *M. parviflora* extracts across various models. The leaf methanol extract exhibited significant anti-inflammatory activity *in vivo*, reducing both acetic acid-induced vascular permeability and croton oil-induced ear edema in mice models^18^. Furthermore, the hexane extract of South African *M. parviflora* leaves had the highest anti-COX-1 activity (42-77%), outperforming both methanol (16-53%) and water (5-40%) extracts^53^. These findings align with our results, which also identify the hexane fraction as the one that has the highest anti-inflammatory potency among the tested fractions. Additionally, an oleanolic acid derivative, isolated from the chloroform extract of *M. parviflora* aerial parts, inhibited both COX-2 and LOX in a diabetic mouse model^54^.

Based on these potent anti-inflammatory effects, comparable to those of standard drugs, our results suggest that *M. parviflora* can be considered a functional food for systemic chronic low-grade inflammation.

### *In vitro* inhibitory activities of cassipourol and *β*-sitosterol against COX-1, COX-2, and 5-LOX

Table 3 illustrates the *in vitro* anti-inflammatory activity of the isolated compounds, cassipourol and *β*-sitosterol, compared with the standard drugs. Cassipourol demonstrated moderate inhibition of COX-1 (38.79% at 100 µg/mL, IC₅₀ = 10.08 µg/mL) and COX-2 (41.04%, IC₅₀ = 7.47 µg/mL), with lower inhibition of 5-LOX (33.89%, IC₅₀ = 11.99 µg/mL). On the other hand, *β*-sitosterol exhibited notable inhibitory activity against COX-1 (59.15%, IC₅₀ = 6.61 µg/mL) and COX-2 (61.40%, IC₅₀ = 4.99 µg/mL). These values were comparable to those of indomethacin (COX-1: 64.67% at 100 µg/mL, IC₅₀ = 6.04 µg/mL; COX-2: 66.92%, IC₅₀ = 4.58 µg/mL). Additionally, *β-*sitosterol inhibited 5-LOX by 54.25% (IC₅₀ = 7.85 µg/mL), with a nonsignificant difference compared with zileuton (51.77%, IC₅₀ = 7.49 µg/mL).

**Table 3:** *In vitro* COX-1, COX-2 and 5-LOX enzyme assays of cassipourol and *β*-sitosterol.

	Anti-inflammatory activity					
	COX-1		COX-2		5-LOX	
	Inhib. (%)	IC_50_ (µg/mL)	Inhib. (%)	IC_50_ (µg/mL)	Inhib. (%)	IC_50_ (µg/mL)
Cassipourol	38.79 ± 0.37^***^	10.08 ± 0.14^***^	41.04 ± 0.37^***^	7.47 ± 0.06^***^	33.89 ± 0.37^***^	11.99 ± 0.12^***^
*β*-Sitosterol	59.15 ± 0.56^***###^	6.61 ± 0.09^*###^	61.40 ± 0.56^***###^	4.99 ± 0.04^**###^	54.25 ± 0.56^*###^	7.85 ± 0.04^###^
Indomethacin	64.67 ± 0.23	6.04 ± 0.05	66.92 ± 0.23	4.58 ± 0.05	–	
Zileuton	–		–		51.77 ± 0.23	7.49 ± 0.07

Values are expressed as mean ± standard error (SE) from three independent replicates. Indomethacin and zileuton were used as reference drugs for COX-1/2 and 5-LOX assays, respectively. Percentage inhibition (Inhib. %) was calculated at a fixed concentration of 100 µg/mL. Statistical significance is indicated as follows: *, *p* ≤ 0.05; **, *p* ≤ 0.01; ***, *p* ≤ 0.001 versus standard drug. ^#^, *p* ≤ 0.05; ^##^, *p* ≤ 0.01; ^###^, *p* ≤ 0.001 versus cassipourol.

Overall, these findings indicate that the isolated compounds exhibit dual COX/LOX inhibitory activity, contributing to the anti-inflammatory potential of *M. parviflora*, with *β*-sitosterol demonstrating stronger activity than cassipourol.

Cassipourol is a monocyclic diterpene alcohol that was originally isolated from *Cassipourea madagascariensis* leaves and roots^52^. Although specific anti-inflammatory studies of cassipourol are currently lacking, its potential activity is suggested by its structural classification and analogy. Its chemical class, diterpenes, is well known for its anti-inflammatory effects^55,56^. Furthermore, its structural analog, phytol (C_20_H_40_O), also demonstrated anti-inflammatory activity^57^, providing a strong basis for investigating the anti-inflammatory potential of cassipourol.

*β*-Sitosterol is an abundant metabolite belonging to phytosterols, which are plant-derived analogs of animal cholesterol that offer numerous health benefits^58^. Since human bodies cannot synthesize them, phytosterols are essential components that must be obtained from the diet^59^. *β*-Sitosterol’s presence in *M. parviflora* is well documented. It was isolated, as a mixture with stigmasterol, from the light petroleum ether fraction of the aerial parts’ ethanol extract^11^, and also identified in the chloroform extract of the root bark^14^. Furthermore, its anti-inflammatory effect has been previously reported. For example, *β*-sitosterol reduced COX-1 and COX-2 expressions by 0.54-fold and 0.36-fold, respectively, in the red swamp crayfish model of white spot syndrome after 24 h of 50 mg/Kg injection^60^. Furthermore, it dose-dependently repressed COX-2 and PGE2 production in A549 cells infected with influenza A virus (at 150–450 µg/mL) after 24 hours^61^.

From a safety perspective, combining COX and LOX inhibition represents a beneficial therapeutic strategy. This dual action counteracts the redirection of arachidonic acid toward the leukotriene pathway, a compensatory side effect observed with selective COX inhibition. Such a balanced approach is critical for augmenting overall anti-inflammatory activity while mitigating leukotriene-associated risks, such as bronchoconstriction and gastric irritation^62^. Furthermore, given the hepatotoxicity of the FDA-approved 5-LOX inhibitor zileuton (due to its thiophene moiety)^31^, both cassipourol and *β*-sitosterol (or their optimized derivatives) represent promising alternatives for 5-LOX inhibition. To the best of our knowledge, we are the first to report the anti-inflammatory potential of cassipourol against COX-1, COX-2, and 5-LOX. Additionally, we confirm the previously reported COX inhibitory activity of *β*-sitosterol and newly demonstrate its inhibitory activity against 5-LOX.

### Network pharmacology

#### Compounds-targets-inflammation network

**Fig. 2:**
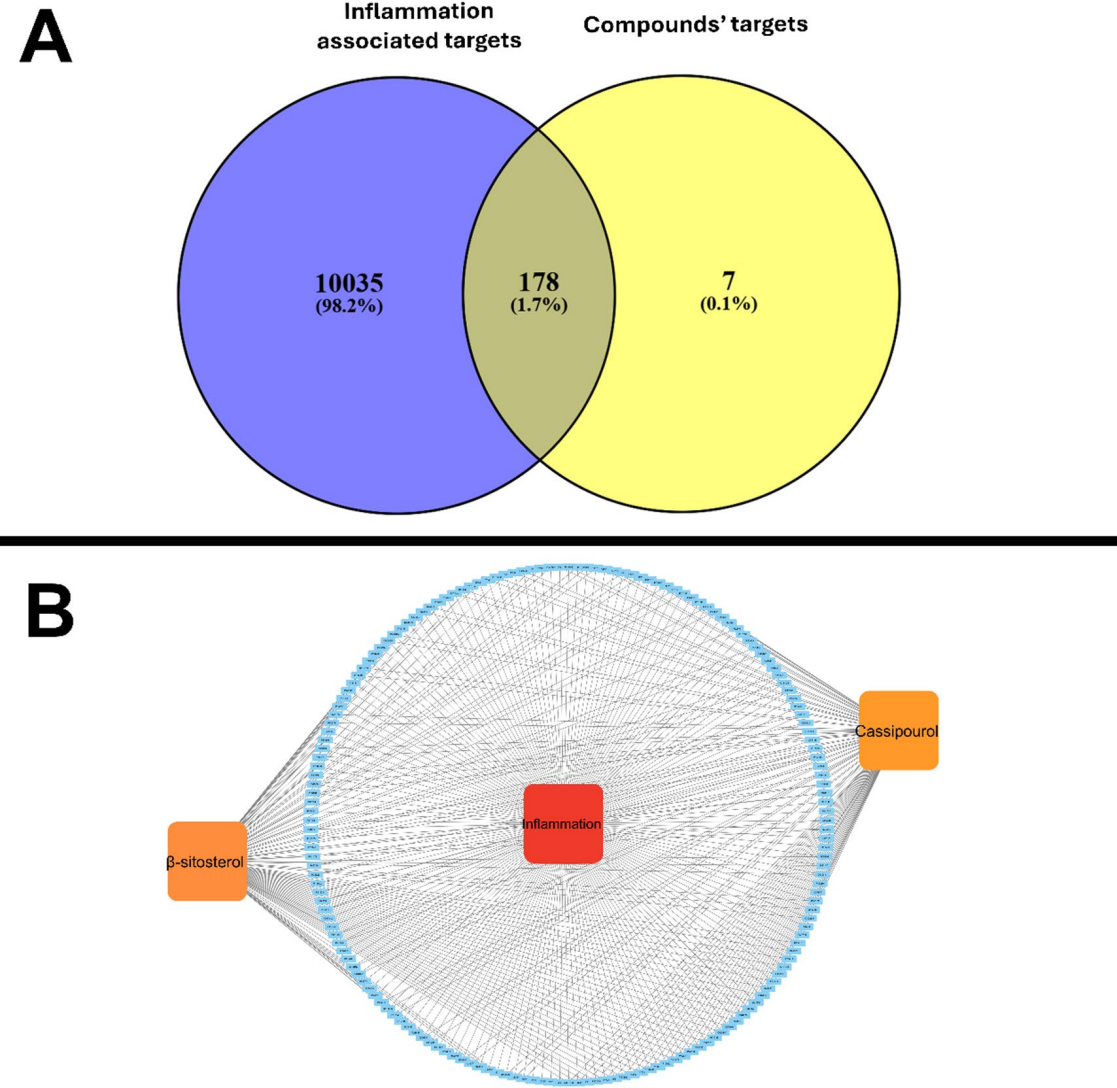
(**A**) Venn diagram representing the intersecting targets of inflammation associated proteins and isolated compounds. (**B**) Compounds-Targets-Inflammation network.

The analysis of the Venn diagram (Fig. 2A) revealed 178 common targets shared by the isolated compounds and inflammation. These common targets are visualized in the Compounds (cassipourol and *β*-sitosterol)-Targets-Inflammation network (Fig. 2B) and are fully listed in (Suppl. Table S5) and ranked by degree. Degree represents the number of connections a node (target) has within the network; thus, targets with a higher degree are considered to possess greater influence^63^.

#### PPI network

**Fig. 3:**
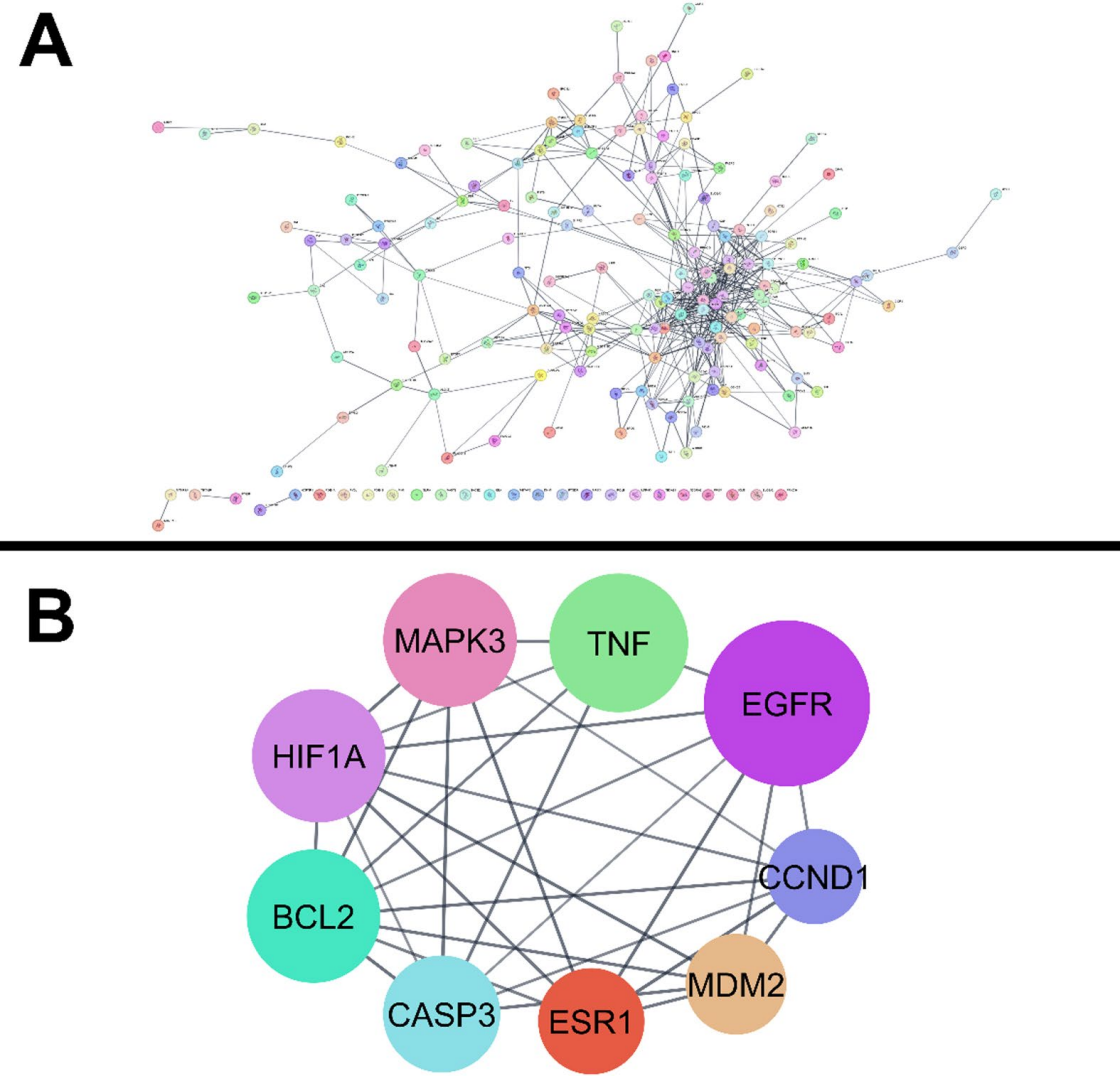
(**A**) PPI network of common targets for inflammation and the two compounds. (**B**) Core targets’ sub-network; Node size in (**B**) corresponds to the degree of interconnection. (EGFR: epidermal growth factor receptor, TNF-α: tumor necrosis factor-alpha, MAPK3: mitogen-activated protein kinase 3, HIF1A: hypoxia inducible factor 1 subunit alpha, BCL-2: B cell lymphoma-2 anti-apoptotic protein, CASP3: Caspase 3, ESR1: Estrogen receptor 1, MDM2: MDM2 proto-oncogene, and CCND1: Cyclin D1).

The PPI network of common targets was constructed using a specialized database that connects targets based on reported functional interactions. As in the compound-target-disease network analysis, a higher degree value in the PPI network indicates greater functional importance of the protein in the disease pathway^32^. The resulting PPI network (Fig. 3A) identified five proteins as key inflammation-related targets for cassipourol and *β*-sitosterol, including epidermal growth factor receptor (EGFR), tumor necrosis factor-alpha (TNF-*α*), mitogen-activated protein kinase 3 (MAPK3), hypoxia inducible factor 1 subunit alpha (HIF1A), and B cell lymphoma-2 antiapoptotic protein (BCL-2). Figure 3B illustrates a subnetwork of the PPI for the top 9 core targets (degree value ≥ 17 nodes), with node size proportional to its degree. All nodes from the PPI network are arranged by degree (Suppl. Table S6). Based on their critical roles, TNF-*α*, as a key player in inflammation pathways^64^, and BCL-2, as a representative for apoptotic proteins^65^, were selected for further *in vitro* studies.

#### GO terms and KEGG pathways enrichment

**Fig. 4:**
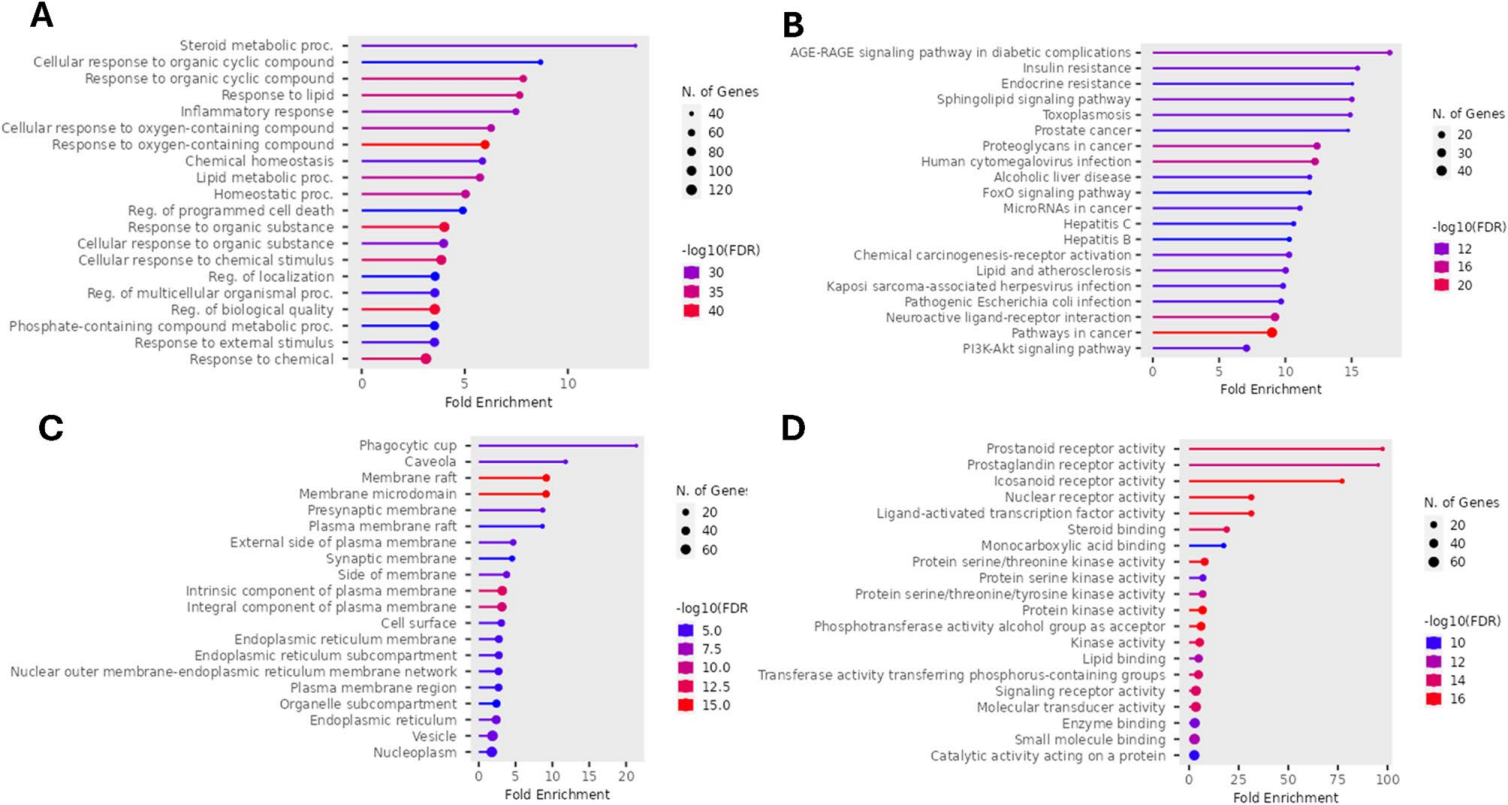
Enrichment analysis of the common targets of inflammation and two compounds. (**A**) Top GO biological processes, (**B**) Top KEGG pathways, (**C**) Top Go cellular components, and (**D**) Top Go molecular functions. The color density and the circle size represent p-value and number of involved genes, respectively.

GO and KEGG fold enrichment were performed to identify the biological pathways associated with the studied targets^32^. The results, illustrated in Fig. 4, highlighted the 20 most significant related functions. The most enriched biological processes (Fig. 4A) include steroid metabolic processes, inflammatory response, responses to oxygen-containing compounds, and regulation of programmed cell death. Regarding the KEGG pathways (Fig. 4B), the most significant pathways included the advanced glycation end products and their receptor (AGE-RAGE) and cancer pathways. The top enriched cellular components (Fig. 4C) were the phagocytic cup, caveola, and membrane raft. Finally, the associated molecular function (Fig. 4D) was enriched in the receptor activity of proinflammatory lipids, including prostanoids, prostaglandins, and icosanoids.

### *In vitro* downregulation activity of cassipourol and *β*-sitosterol against TNF-*α* and BCL-2 activity

Network pharmacology analyses of cassipourol and *β*-sitosterol identified additional inflammatory targets, with TNF-*α* and BCL-2 selected for subsequent experimental validation. TNF-*α* is an inflammatory cytokine that is overexpressed in higher-stage colorectal cancers; it induces an inflammatory colorectal cancer microenvironment by dysregulating the immune response and enhancing cancer cell invasion and migration, among other effects^66^. Additionally, TNF-*α* participates in non-small cell lung cancer (NSCLC) resistance to targeted treatment through activation of the proinflammatory transcription nuclear factor kappa-B (NF-kB)^67^. BCL-2 is an antiapoptotic protein whose overproduction inhibits apoptosis in cancer and inflammatory cells, fostering different types of cancers (i.e., colorectal and lung cancers)^68,69^ and inflammatory disorders such as particulate matter-induced lung inflammation^70^. Therefore, nonsteroidal anti-inflammatory drugs (NSAIDs) attracted research attention to direct their anti-inflammatory and apoptosis induction activities against various cancers^71,72^. For example, indomethacin, particularly in combination with juglone (a natural naphthoquinone derivative), attenuated cancer cells’ survival in human colon adenocarcinoma (HT29) cell line by suppressing TNF-*α*, NF-kB, COX-2, and BCL-2, while elevating a group of proapoptotic proteins^43^. Hence, due to the strong enrichment of cancer pathways in our network pharmacology analysis, we further tested the isolated compounds using Caco-2 and A549 cell lines as a novel application.

Table 4 summarizes the results for both compounds. Compared with DMSO as a control, cassipourol showed a weak reduction in both targets (~20%), with IC₅₀ values of 222.27 and 89.84 µg/mL in Caco-2 and A549 cells, respectively. In contrast, *β*-sitosterol (IC₅₀ = 177.73 and 53.94 µg/mL) and indomethacin (standard drug; IC₅₀ = 35.25 and 25.73 µg/mL) exhibited strong downregulation against the two targets. *β*-Sitosterol reduced both proteins at approximately 55% and indomethacin at 85%.

**Table 4:** Cassipourol and *β*-sitosterol downreguation effect on TNF-*α* and BCL-2 in human colon carcinoma (Caco-2) and lung cancer (A549) cells lines.

	Caco-2		A549	
	TNF-α (Pg/mL)	BCL-2 (ng/mL)	TNF-α (Pg/mL)	BCL-2 (ng/mL)
DMSO (control)	333.59 ± 1.00^a^	16.15 ± 0.15^a^	407.75 ± 1.26^a^	13.15 ± 0.10^a^
Cassipourol	266.87 ± 0.80^ab^	12.92 ± 0.12^ab^	326.20 ± 1.01^ab^	10.52 ± 0.08^ab^
*β*-Sitosterol	148.26 ± 0.44^abc^	7.18 ± 0.07^abc^	181.22 ± 0.56^abc^	5.84 ± 0.04^abc^
Indomethacin	49.51 ± 0.15	2.40 ± 0.02	60.52 ± 0.19	1.95 ± 0.01

Values are presented as mean ± standard error (SE) from three independent replicates. Indomethacin was used as the reference anti-inflammatory drug. In Caco-2 cells, treatments were applied at their respective IC₅₀ values: Cassipourol (222.27 µg/mL), *β*-sitosterol (177.73 µg/mL), and indomethacin (35.25 µg/mL). In A549 cells, the corresponding IC₅₀ values were 89.84, 53.94, and 25.73 µg/mL, respectively. Statistical significance is indicated as follows: ^a^, significantly different from the standard; ^b^, significantly different from DMSO-treated control cells; and ^c^, significantly different from cassipourol-treated cells (*p* ≤ 0.05).

Previous studies have demonstrated *β*-sitosterol’s anti-inflammatory and cytotoxic activities. Specifically, concentrations of 8 and 16 µM reduced the lipopolysaccharides (LPS)-induced TNF-*α* and other inflammatory markers by about 50 and 75%, respectively, in a murine microglial (BV2) cell line^73^. Furthermore, the cytotoxic effect of the *Grewia tiliaefolia* leaves’ benzene extract against A549 cells was linked to the downregulation of BCL-2 and the regulation of other apoptotic proteins. Subsequent cytotoxicity-guided fractionation confirmed that the isolated *β*-sitosterol had cytotoxicity against the same cell line^74^. It is noteworthy that the partial downregulation of TNF-*α* by *β*-sitosterol could be an advantage if used as an adjuvant with other anticancer drugs, as TNF-*α* is known to boost the vascular permeability, enhancing the accumulation of anticancer treatments inside tumor cells^75^.

Hence, our results clearly position *β*-sitosterol as a potential drug candidate or adjuvant therapy for inflammatory disorders and cancers, particularly those affecting the lungs, due to its proven anti-inflammatory and apoptotic-inducing activities. Furthermore, anti-TNF drugs for other peripheral autoimmune disorders, including rheumatoid arthritis and Crohn’s disease, are associated with neurological adverse effects such as neuronal demethylation and lesions^76^. Therefore, based on our observations, besides the reported neuroprotective activity of *β*-sitosterol^77^, we recommend conducting future studies about *β*-sitosterol as a substitute for typical anti-TNFs against peripheral autoimmune diseases in patients with personal or family history of neurological disorders.

Regarding cassipourol, its weak activity against TNF-*α* suggests it could be uniquely beneficial for conditions like multiple sclerosis (MS) and should be tested in future studies. This chronic autoimmune disorder is characterized by elevated levels of TNF-*α*, COX-1, COX-2, and 5-LOX, inflammatory mediators^78,79^. However, studies reported that anti-TNF-*α* therapy unexpectedly aggravated MS condition and increased relapses^79^. To our knowledge, this is the first study to investigate the activity of cassipourol against the inflammatory mediators TNF-*α* and BCL-2.

### Molecular docking

*In silico* molecular docking is a rapid technique for predicting the inhibitory potential of specific compounds against target proteins^80^. In this study, we employed molecular docking simulations to assess the inhibitory activities of cassipourol and *β*-sitosterol against COX-1, COX-2, 5-LOX, TNF-*α*, and BCL-2. The results (Table 5; Suppl. Figs. S10:S14) revealed the key interactions and displayed comparable docking scores to the reference standards.

**Table 5:** Binding scores and active sites interactions of inflammatory targets with cassipourol and *β*-sitosterol.

	Binding score and interacted amino acids									
	COX-1		COX-2		5-LOX		TNF-α		BCL-2	
	Score	Interactions	Score	Interactions	Score	Interactions	Score	Interactions	Score	Interactions
Cassipourol	–4.4	Tyr 55	–6.58	His 214	–6.0	Arg 596, His 600	–4.96	Tyr 59	–5.67	Tyr 108, Arg 146
*β*-Sitosterol	–4.85	Tyr 38, Asp 53	–6.3	Asn 382	–5.3	Arg 596, His 600	–5.24	Tyr 59	–4.81	Tyr 108, Arg 146
Indomethacin	–4.32	Lys 468	–6.8	Ala 202, Leu 391	–		–4.75	Tyr 59	–6.38	Glu 136, Arg 146
Zileuton	–		–		-5.3	Phe 359, His 360, Thr 364	–		–	

Indomethacin was used as the reference ligand for COXs, TNF-*α*, and BCL-2, while zileuton was employed as the reference ligand for 5-LOX.

The binding scores of cassipourol (–4.4 and −6.58) and *β*-sitosterol (–4.85 and −6.3) to COX-1 and COX-2 active sites, respectively, are competitive with indomethacin’s binding scores (–4.32 and −6.8). For 5-LOX, the scores of cassipourol (–6) and *β*-sitosterol (–5.3) are similarly aligned with the zileuton standard docking score (–5.3). Against TNF-*α* and BCL-2, the docking scores for cassipourol (–4.96 and −5.67) and *β*-sitosterol (-5.24 and −4.81) are comparable to indomethacin’s scores (–4.75 for TNF-*α* and − 6.38 for BCL-2).

The observed modest cellular activity of cassipourol against TNF-*α* and BCL-2 (20% suppression in Caco-2 and A549 cell lines), despite its notable *in silico* activity, is primarily attributable to its unfavorable physicochemical properties, particularly its high lipophilicity. Computational estimates (XLogP3-AA ≈ 7.5; consensus CLogP ≈ 6.8) place cassipourol well above the optimal lipophilicity threshold (Log *P* > 5) for drug-like molecules^81^. Such excessive hydrophobicity typically compromises aqueous solubility, promotes nonspecific binding to serum proteins and cellular membranes, and limits the freely available intracellular concentration of the compound. Consequently, despite its intrinsic binding potential, cassipourol is likely sequestered in lipid bilayers, reducing its effective delivery to cytosolic or nuclear targets like TNF-*α* and BCL-2. This phenomenon underscores a well-recognized limitation of molecular docking; it evaluates ligand–protein interactions in a static, solvated environment while ignoring pharmacokinetic barriers, such as cellular uptake, membrane permeation, and subcellular distribution^82^. Therefore, the weak suppression of TNF-*α* and BCL-2 (~20%) observed *in vitro* is due to poor bioavailability at the site of action, not a lack of target affinity. Consequently, while cassipourol represents a promising chemical scaffold with genuine multitarget anti-inflammatory potential, its therapeutic applicability may require structural optimization, such as introduction of polar functional groups (e.g., esters, carboxylic acids, or heterocycles) to reduce Log P, improve solubility, and enhance intracellular bioavailability without compromising target engagement^83^. Alternatively, the use of nanosystems and nanotechnology, combined with an appropriate route of administration and an optimized dosing regimen, may markedly enhance its bioavailability^84^.

## Conclusion

This study supports the ethnopharmacological use of *M. parviflora* L. for the treatment of inflammatory disorders by confirming dual COX/LOX inhibition in its extract and fractions. It also reports cassipourol (first identified in the Malvaceae family) and *β*-sitosterol as bioactive anti-inflammatory metabolites contributing to the overall activity of the plant extract. Although *β*-sitosterol is widely distributed in nature, its co-occurrence with cassipourol in this matrix may explain the enhanced activity observed in the hexane fraction, suggesting possible additive or synergistic effects.

These findings extend previous metabolite profiling studies of the plant^12^. Using an integrated *in vitro* and *in silico* approach, *β*-sitosterol was identified as a multitarget anti-inflammatory candidate acting on COX-1/2, 5-LOX, TNF-*α*, and BCL-2, supporting its potential as a therapeutic adjuvant for inflammatory diseases and cancer. Cassipourol’s modest cellular activity in TNF-*α* and BCL-2–related assays, despite its considerable *in silico* activity, may reflect limited cellular exposure or pharmacokinetic constraints rather than a lack of intrinsic target activity. This is supported by the compound’s notable inhibition of COX and 5-LOX in enzyme-based assays, indicating preserved direct enzymatic activity and encouraging further optimization studies to overcome potential pharmacokinetic limitations. Overall, these compounds warrant further investigation. Future work should include *in vivo* pharmacological and pharmacokinetic studies to confirm efficacy, bioavailability, and safety. For cassipourol, structural modifications or formulation strategies may improve bioavailability while preserving its unique low anti-TNF-*α* activity profile for applications in neuroinflammatory diseases such as Multiple Sclerosis, where strong TNF inhibition is contraindicated.

## Supplementary Information

Below is the link to the electronic supplementary material.

Supplementary Material 1

## Data Availability

The authors declare that the data supporting the findings of this study are available within the paper and its Supplementary Data file. Should any raw data files be needed in another format, they are available from the corresponding author upon reasonable request.
