# Supplementary material for: Cassipourol and β-sitosterol from Malva parviflora L.: a mechanistic study of dual anti-inflammatory action against COX/LOX and TNF-α/BCL-2
Source: Sci Rep. 2026 Jun 13;16:18368. doi: 10.1038/s41598-026-56631-1 (PMC13264612; doi:10.1038/s41598-026-56631-1)
Supplement: Supplementary file 1 — Supplementary Material 1 [file 41598_2026_56631_MOESM1_ESM.pdf]

Cassipourol and *β*-Sitosterol from *Malva parviflora* L.: A Mechanistic Study of Dual Anti-Inflammatory Action Against COX/LOX and TNF-*α* /BCL-2

Supplementary Data

Mohamed A. Anwar[1*], Rania A. El Gedaily[1], Wael M. Aboulthana[2], Ahmed Elshewy[3,4]

Zeinab A. Kandil[1], Shymaa I.A. Abdel-dayem[1]

[1]Pharmacognosy Department, Faculty of Pharmacy, Cairo University, Kasr El Aini St., P.B. 11562 Cairo, Egypt

[2]Biochemistry Department, Biotechnology Research Institute, National Research Centre, 33 El Bohouth St., P.O. 12622, Dokki, Giza, Egypt

[3]Pharmaceutical Organic Chemistry Department, Faculty of Pharmacy, Cairo University, Kasr El Aini St., P.B. 11562 Cairo, Egypt

[4]Natural and Applied Sciences Department, College of Arts and Sciences, The American University of Iraq-Baghdad (AUIB), Baghdad, Iraq

Authors' e-mail address:

mohamed.anwar@pharma.cu.edu.eg (Mohamed A. Anwar), rania.elgedaily@pharma.cu.edu.eg (Rania A. El Gedaily), wmkamel83@hotmail.com (Wael M. Aboulthana), ahmed.elshewy@pharma.cu.edu.eg (Ahmed Elshewy), zeinab.kandil@pharma.cu.edu.eg (Zeinab A. Kandil), shymaa.aly@pharma.cu.edu.eg (Shymaa I.A. Abdel-dayem).

***Corresponding author:**

Mohamed A. Anwar

Pharmacognosy Department, Faculty of Pharmacy, Cairo University, Kasr El Aini St., P.B. 11562 Cairo, Egypt

E-mail address: mohamed.anwar@pharma.cu.edu.eg

Tel: +20-1280050639

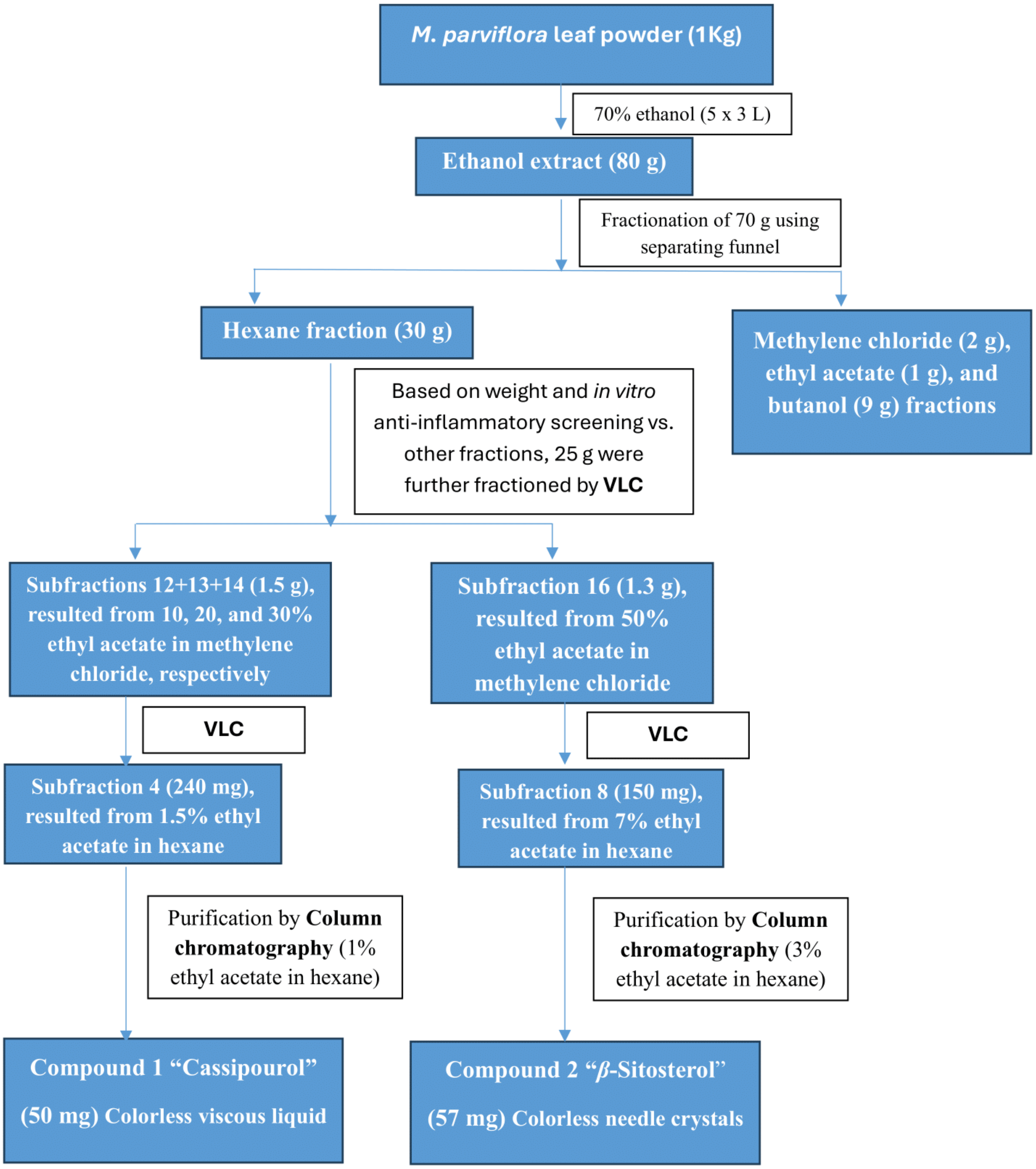

Suppl. Fig. S1. Schematic representation of the steps of extraction, fractionation, and isolation of cassipourol and *β*-sitosterol from *M. parviflora* leaf ethanol extract

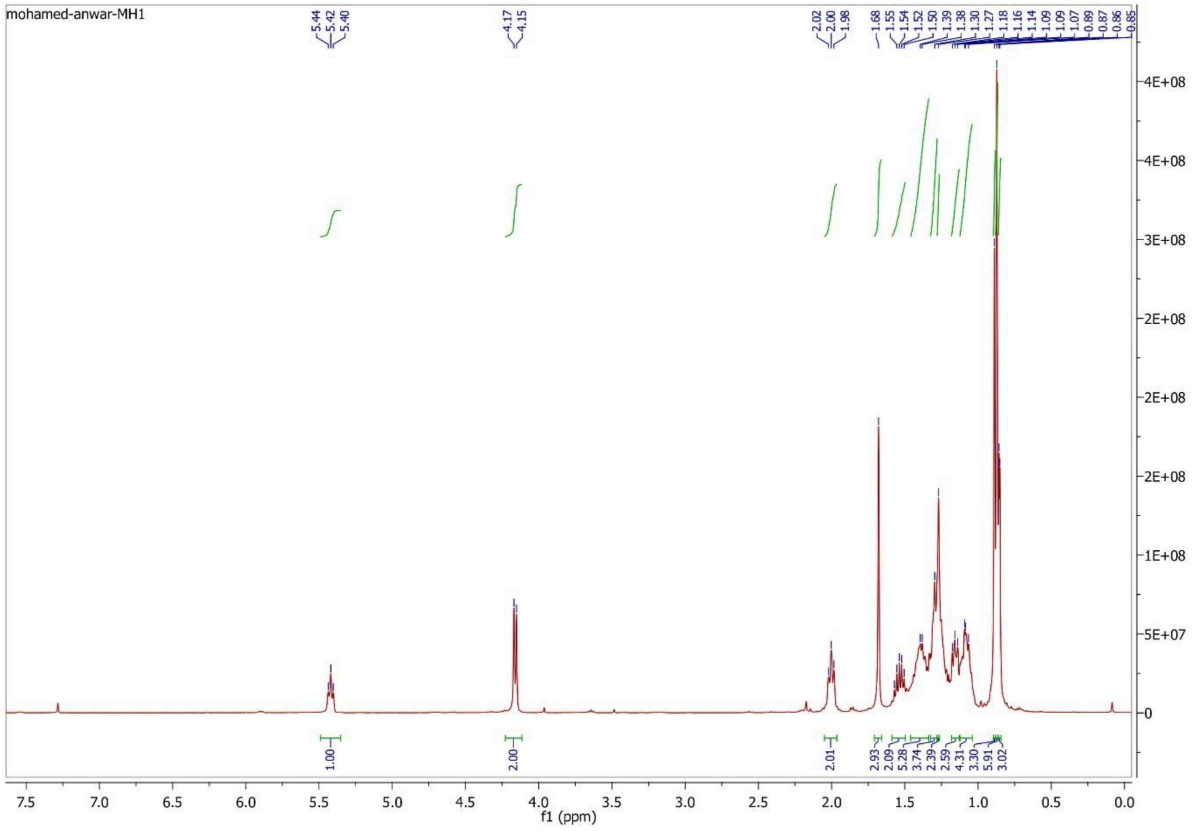

Suppl. Fig. S2. ^{1}H NMR chart of compound 1

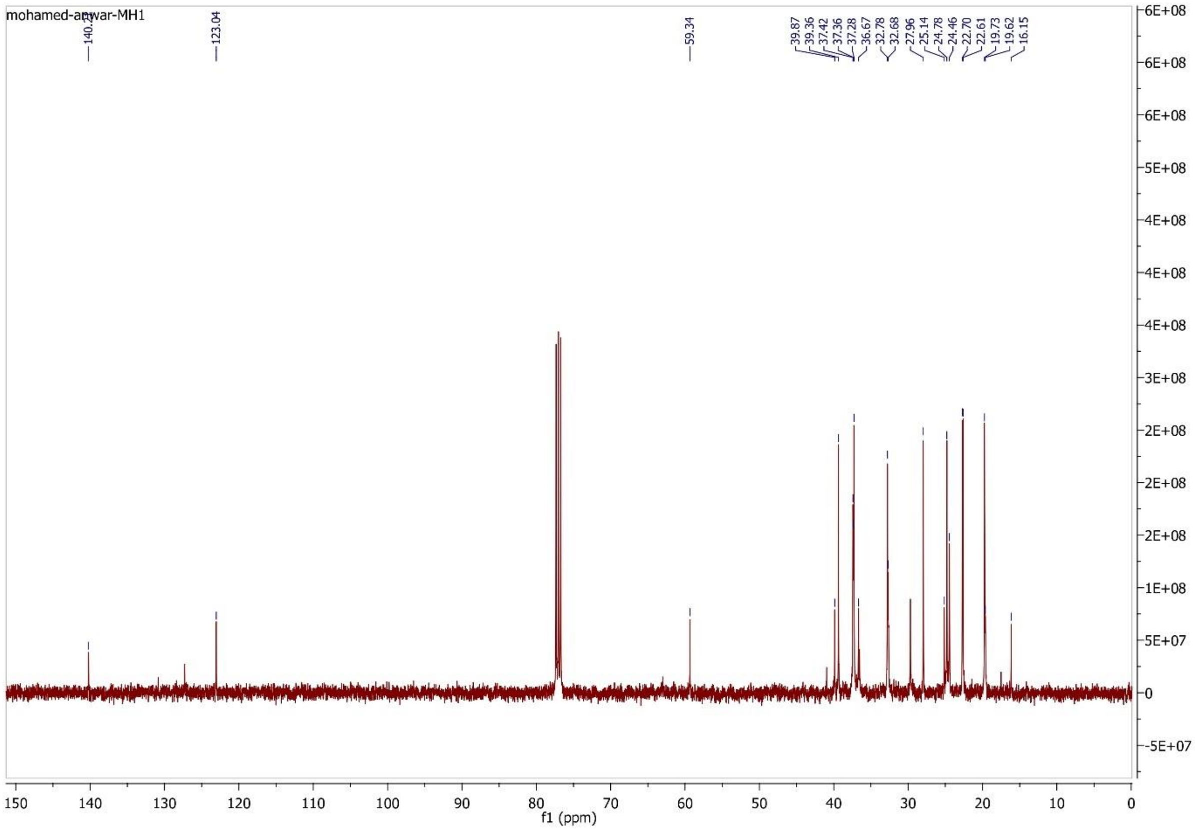

Suppl. Fig. S3. ^{13}C NMR chart of compound 1

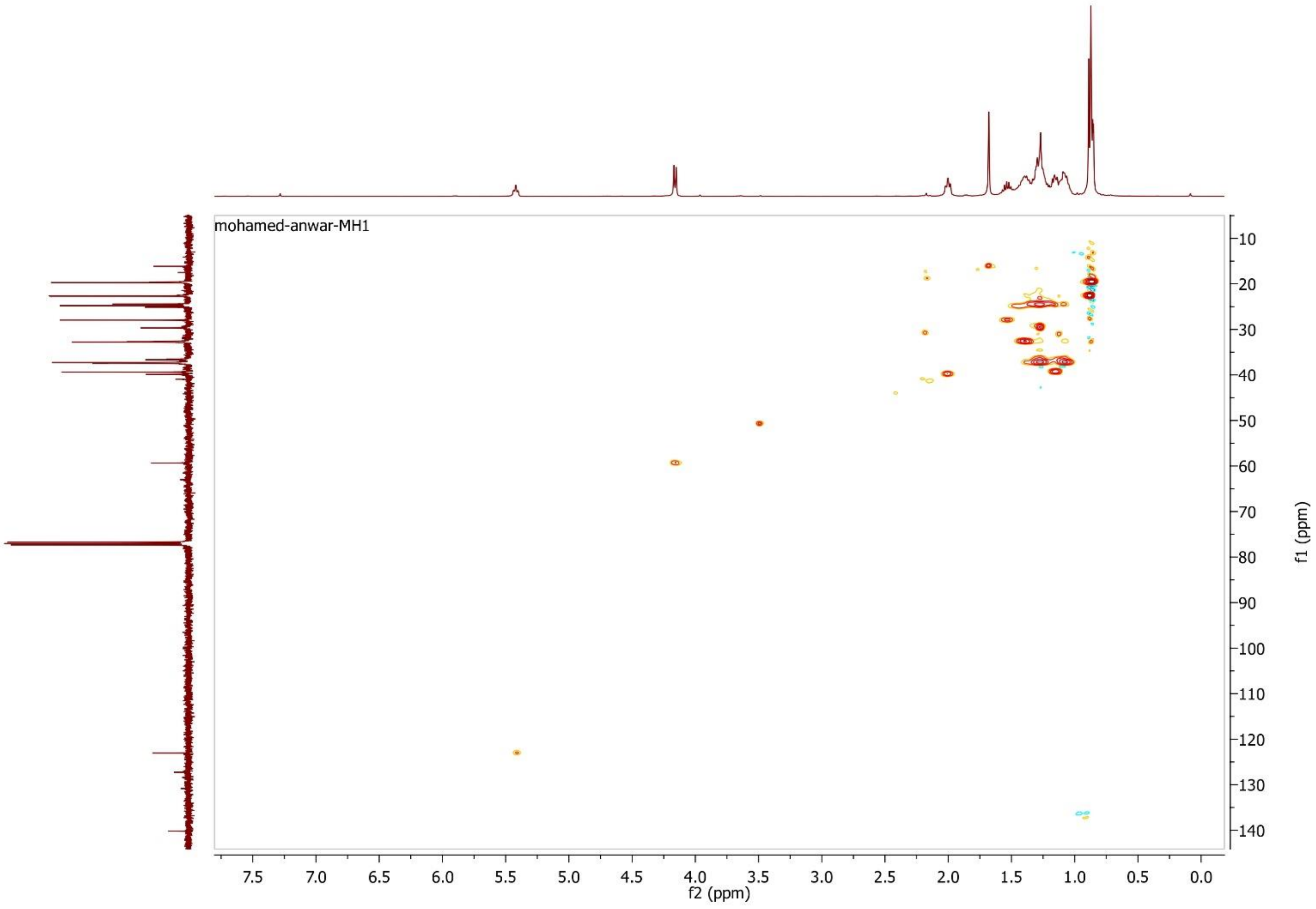

Suppl. Fig. S4. HSQC spectrum of compound 1

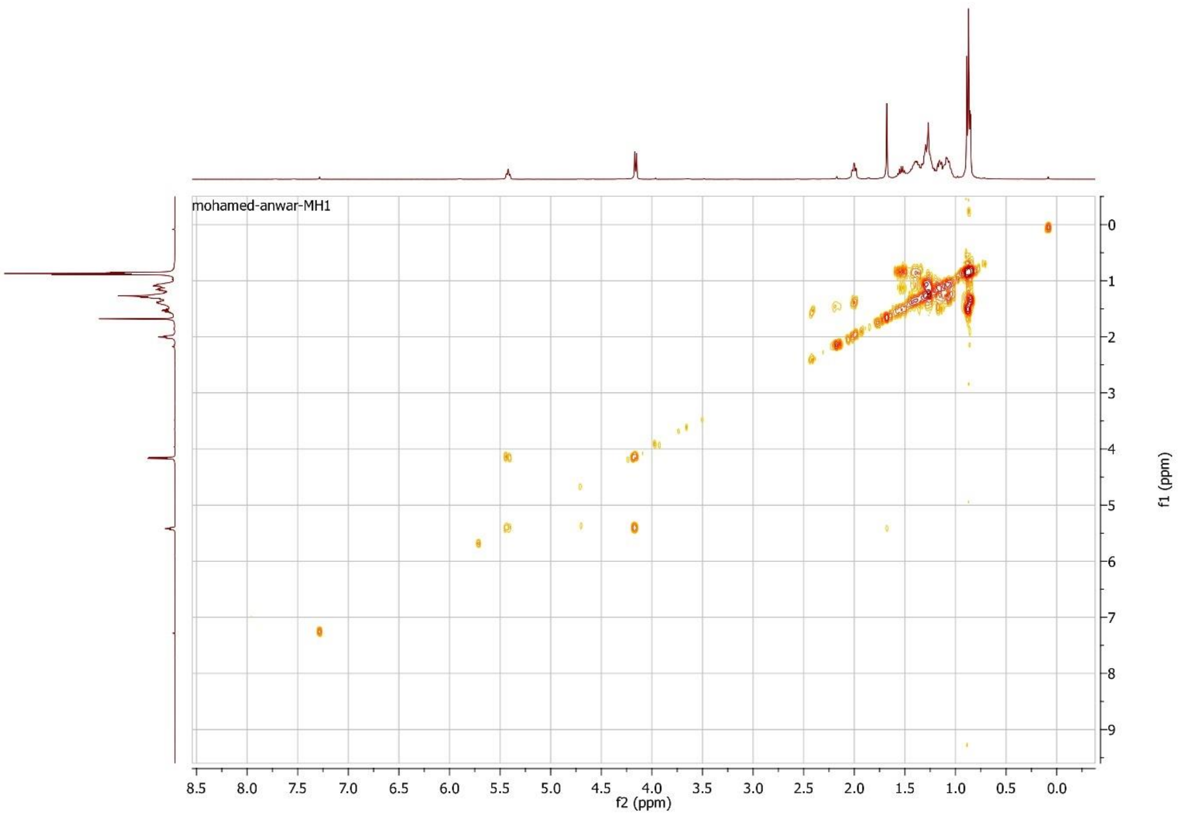

Suppl. Fig. S5. COSY spectrum of compound 1

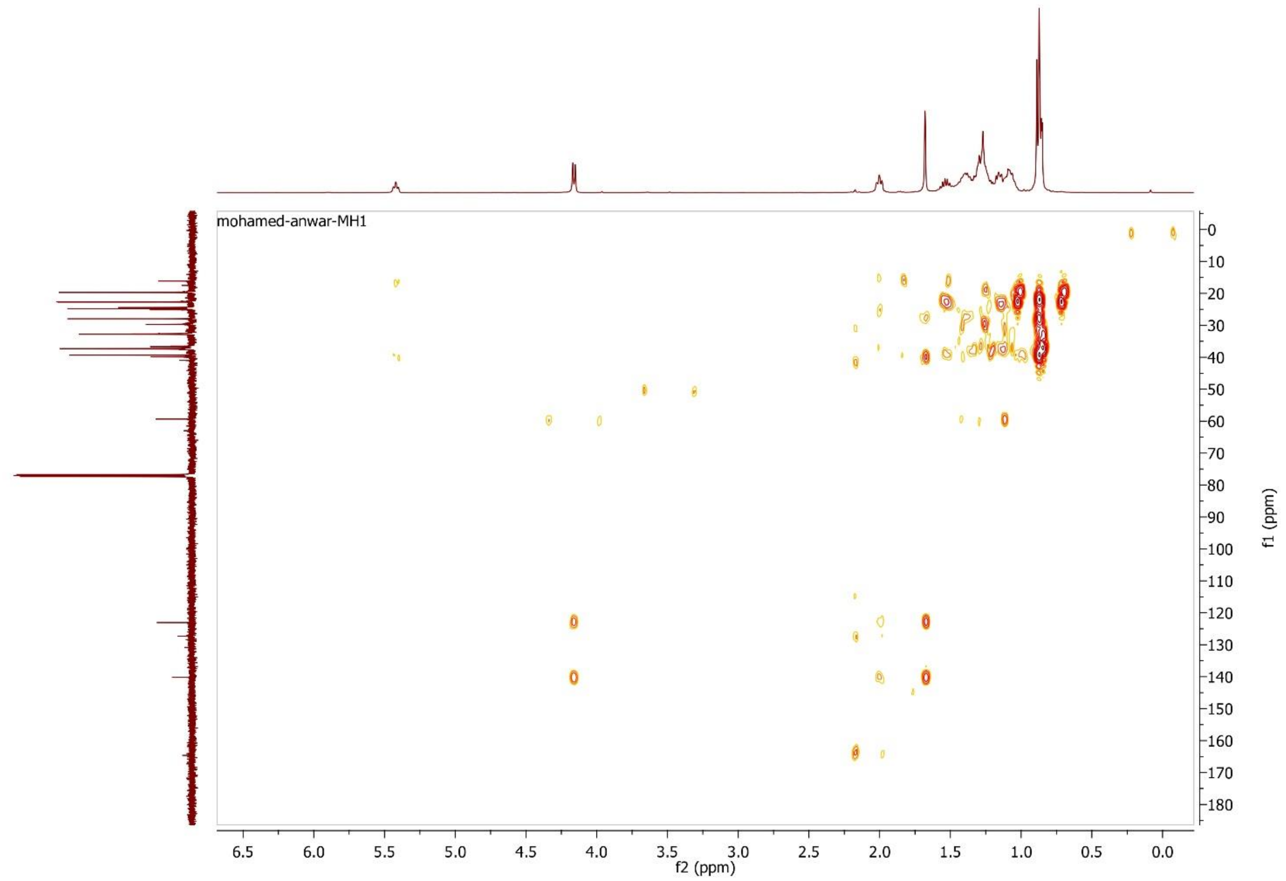

Suppl. Fig. S6. HMBC spectrum of compound 1

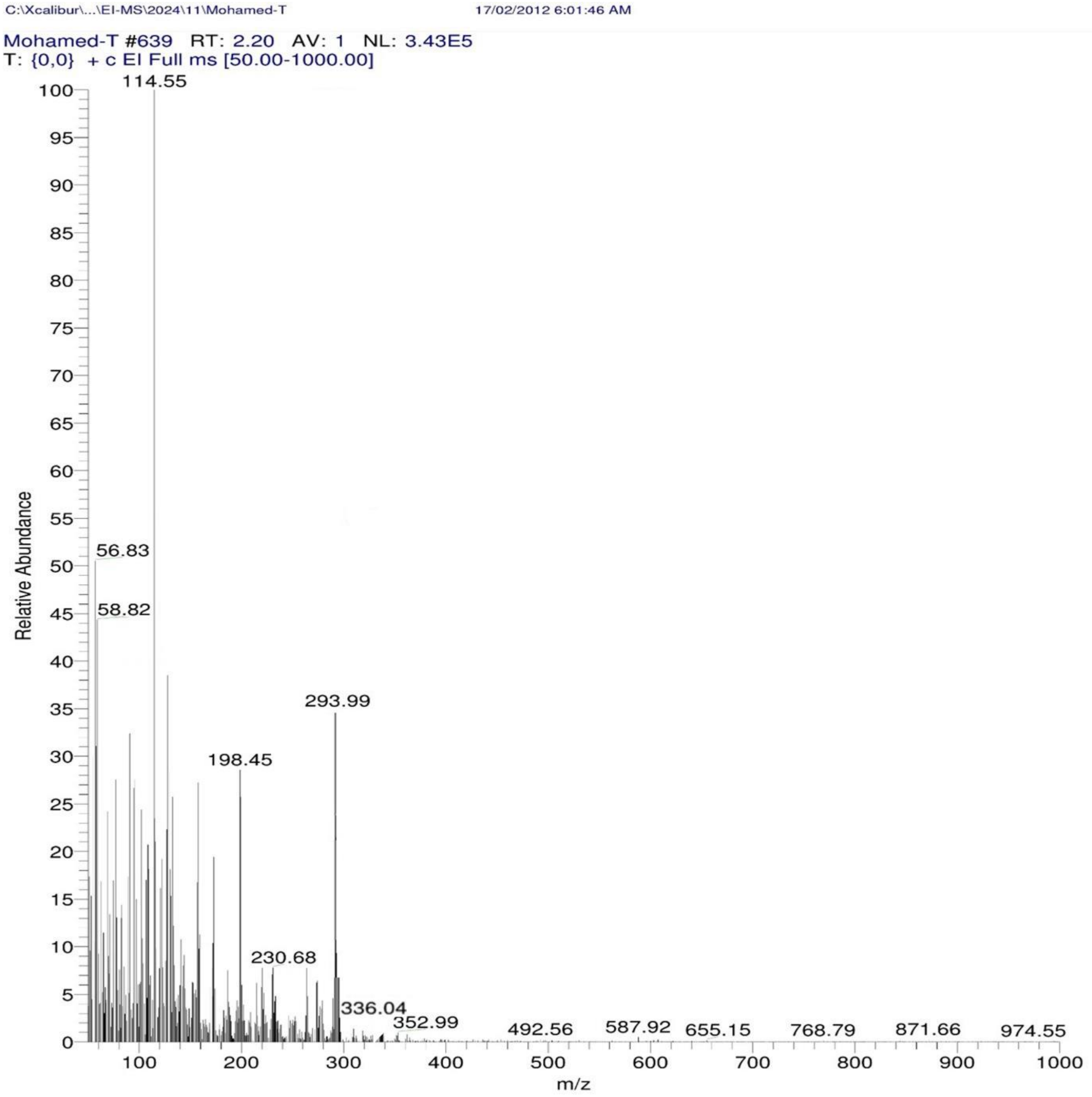

Suppl. Fig. S7. EI-MS mass spectrum of compound 1

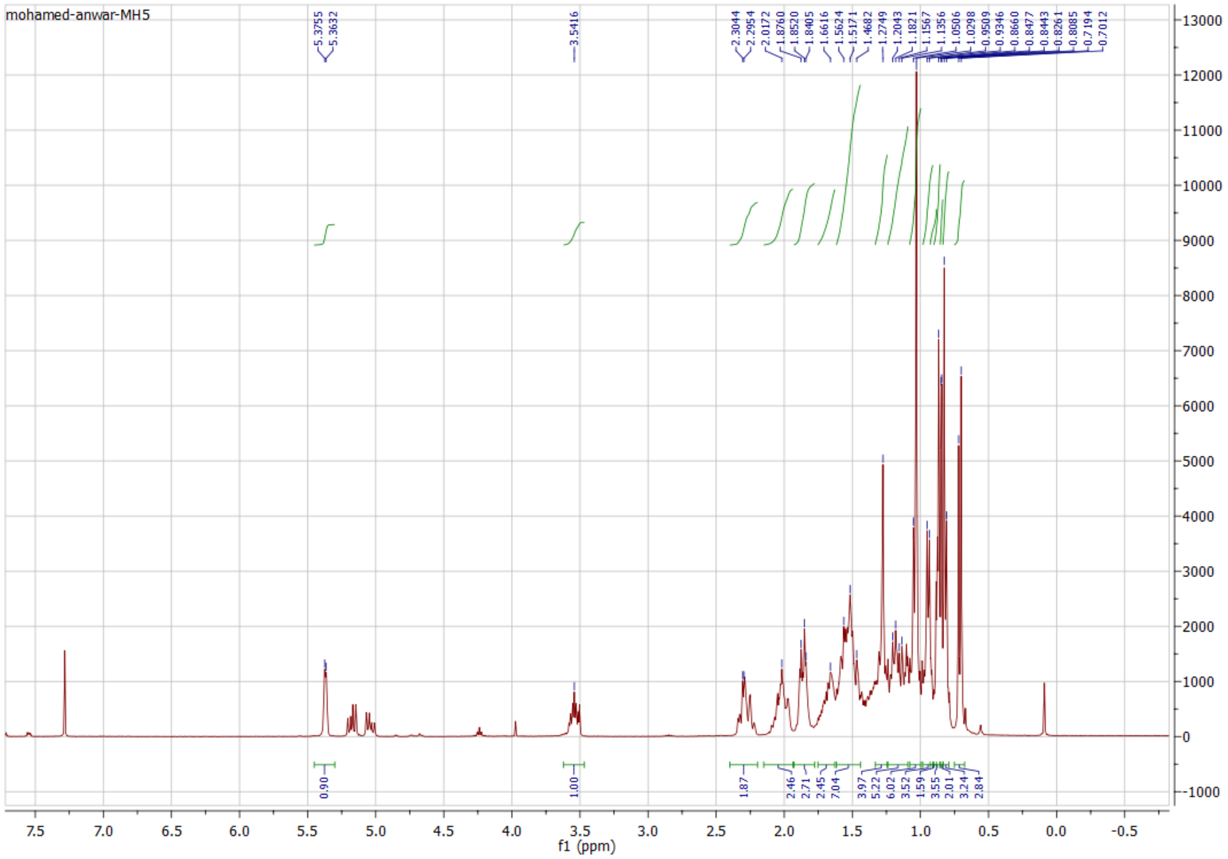

Suppl. Fig. S8. ^{1}H NMR chart of compound 2

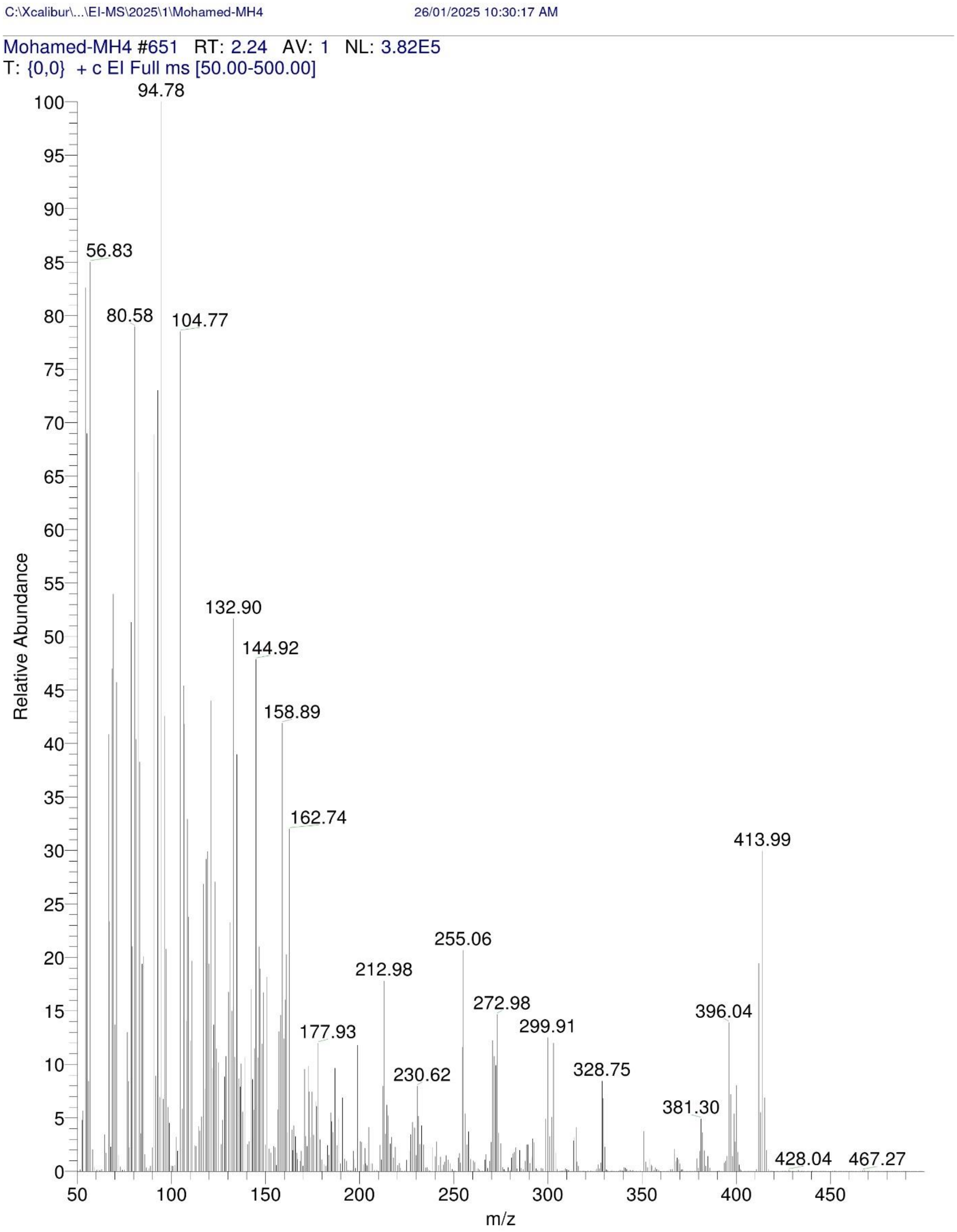

Suppl. Fig. S9. EI-MS mass spectrum of compound 2

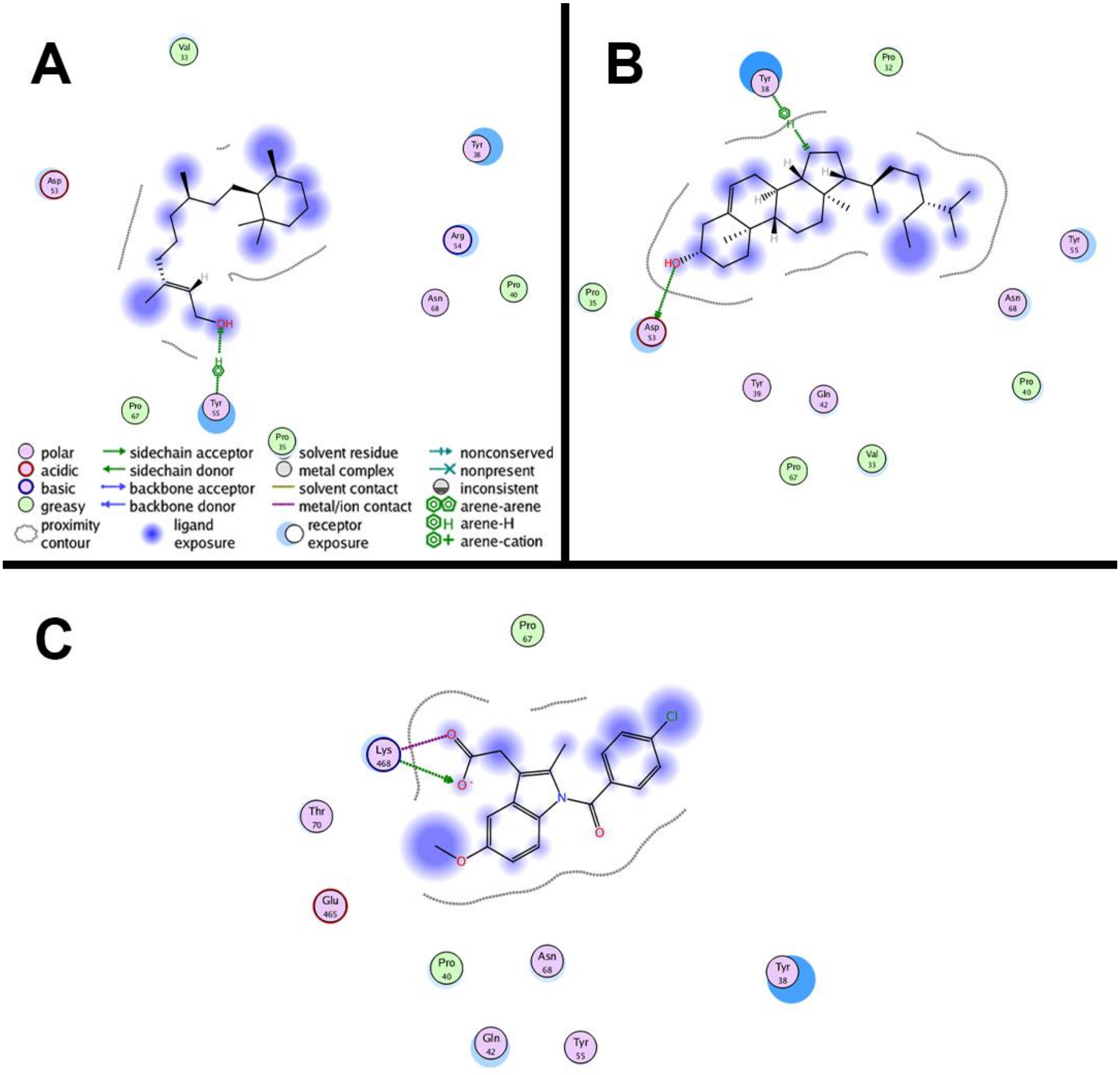

Suppl. Fig. S10. 2D representation of the interactions of (A) cassipourol, (B) *β*-sitosterol and (C) indomethacin with COX-1

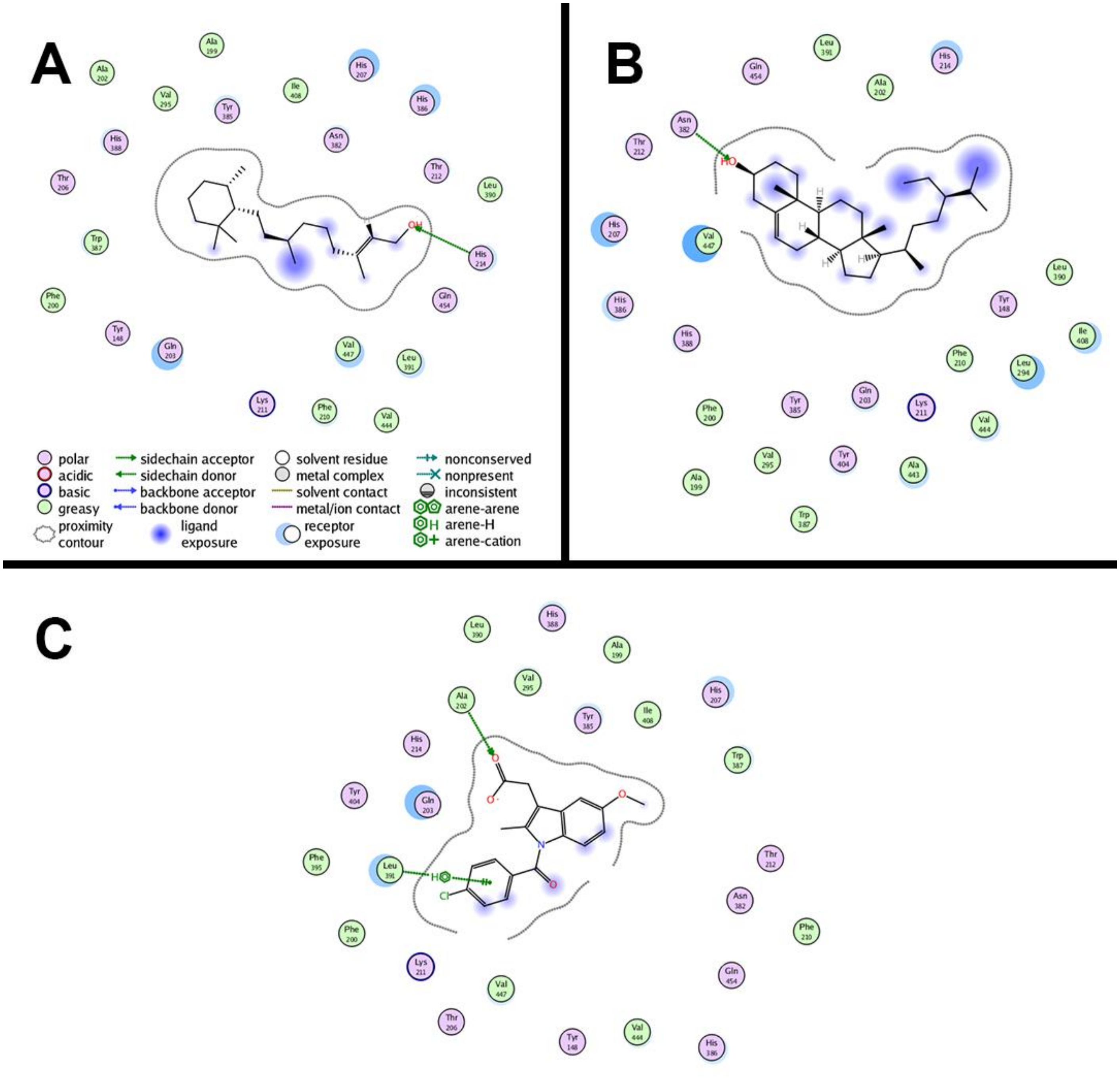

Suppl. Fig. S11. 2D representation of the interactions of (A) cassipourol, (B) *β*-sitosterol and (C) indomethacin with COX-2

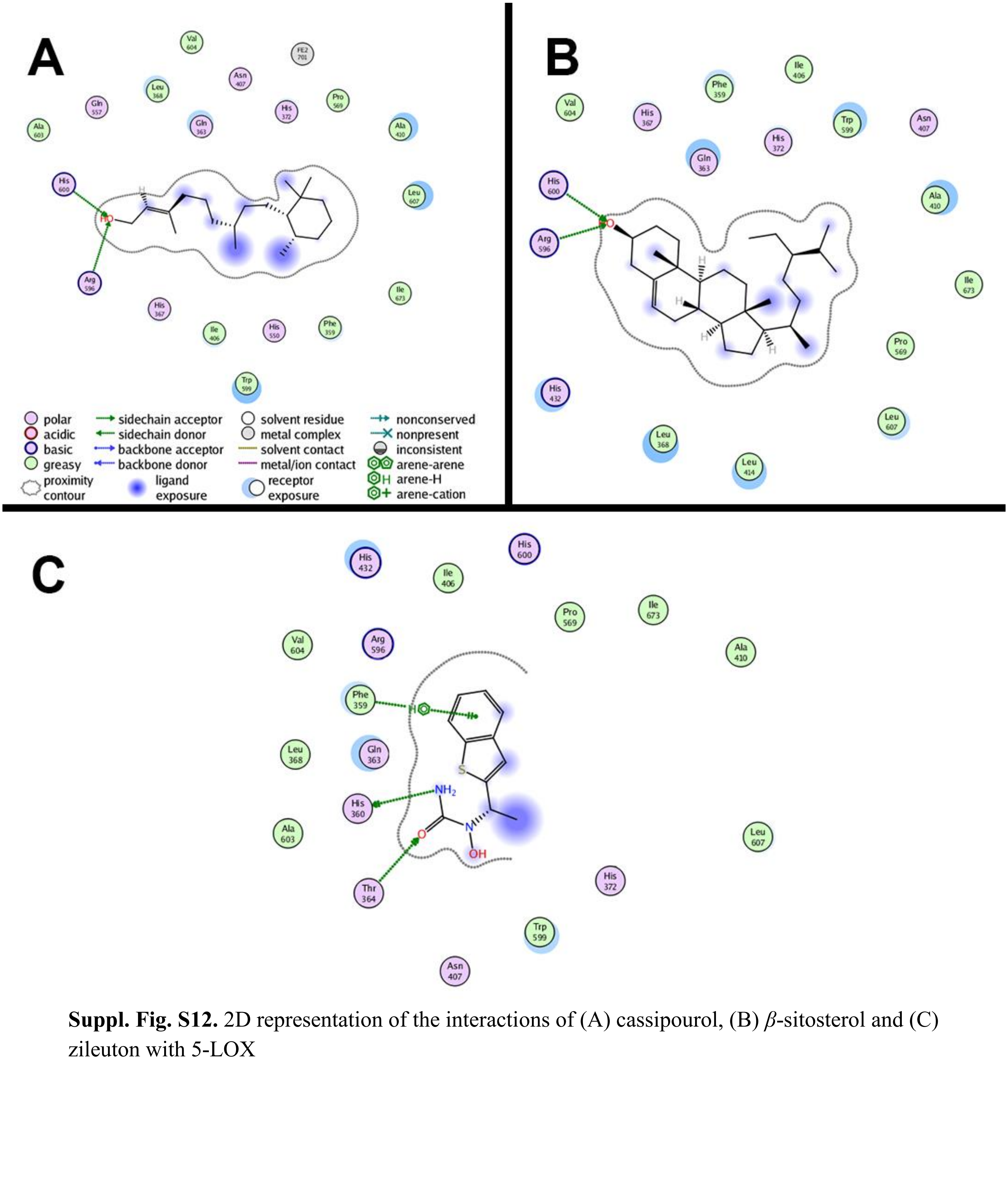

Suppl. Fig. S12. 2D representation of the interactions of (A) cassipourol, (B) *β*-sitosterol and (C) zileuton with 5-LOX

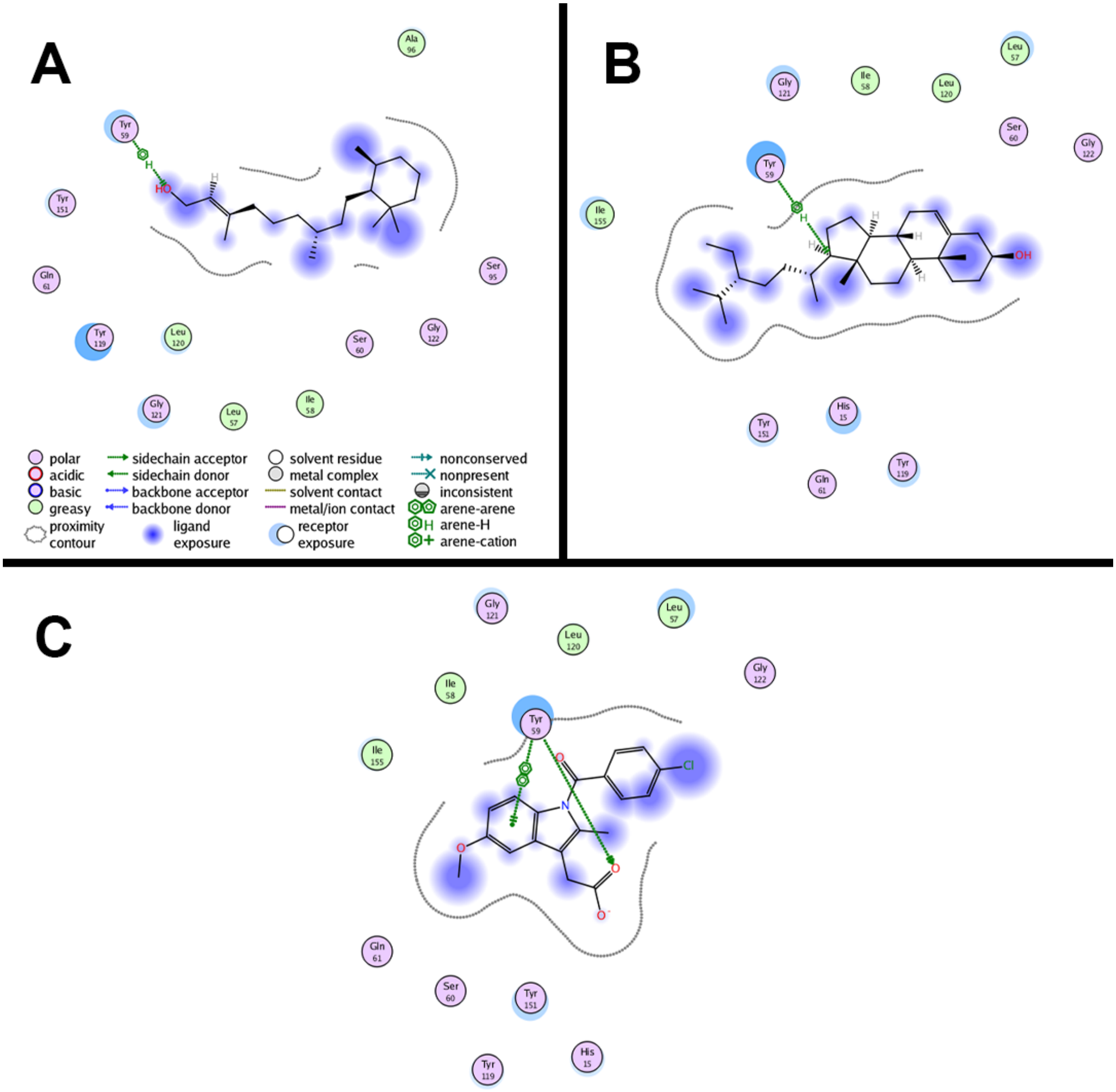

Suppl. Fig. S13. 2D representation of the interactions of (A) cassipourol, (B) *β*-sitosterol and (C) indomethacin with TNF-*α*

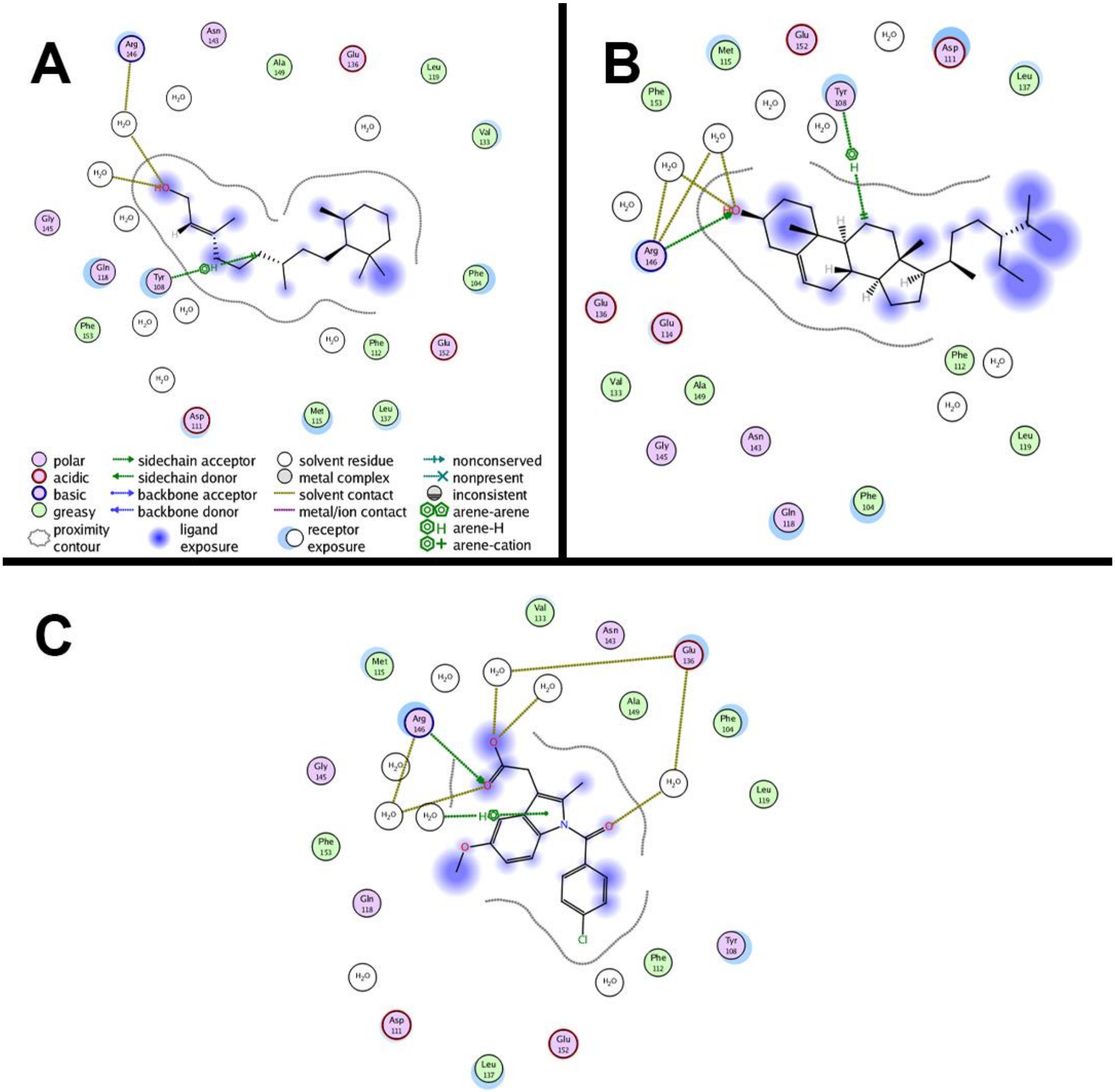

Suppl. Fig. S14. 2D representations of the interactions of (A) cassipourol, (B) *β*-sitosterol and (C) indomethacin with BCL-2

Suppl. Table S1

Raw data for anti-inflammatory IC_{50} values of *M. parviflora* extract and fractions

			Total Ext.	Hex	Methylene chloride	Ethyl acetate	Butanol	STD
Anti-Inflammatory Activity	COX-1	1	6.77	8.48	14.70	31.07	24.66	6.61
		2	6.60	8.27	14.34	30.30	24.05	6.53
		3	6.50	8.14	14.11	29.80	23.66	6.59
		**Mean**	**6.63**	**8.30**	**14.38**	**30.39**	**24.13**	**6.58**
		**SD**	**0.14**	**0.17**	**0.30**	**0.64**	**0.50**	**0.04**
		**SE**	**0.08**	**0.10**	**0.17**	**0.37**	**0.29**	**0.02**
	COX-2	1	5.00	6.21	10.42	20.31	16.62	4.89
		2	5.01	6.22	10.44	20.36	16.66	4.96
		3	4.79	5.95	9.99	19.51	15.96	4.85
		**Mean**	**4.93**	**6.12**	**10.28**	**20.06**	**16.41**	**4.90**
		**SD**	**0.12**	**0.15**	**0.25**	**0.48**	**0.40**	**0.05**
		**SE**	**0.07**	**0.09**	**0.15**	**0.28**	**0.23**	**0.03**
	5-LOX	1	7.30	9.36	17.70	49.25	34.79	8.29
		2	7.26	9.30	17.57	48.71	34.46	8.36
		3	6.96	8.91	16.79	46.12	32.77	8.23
		**Mean**	**7.17**	**9.19**	**17.36**	**48.03**	**34.00**	**8.30**
		**SD**	**0.19**	**0.24**	**0.49**	**1.67**	**1.08**	**0.06**
		**SE**	**0.11**	**0.14**	**0.28**	**0.97**	**0.63**	**0.04**

Indomethacin and zileuton are reference standards (STD) for COXs and LOX, respectively.

Suppl. Table S2

Raw data for anti-inflammatory IC_{50} values for cassipourol and *β*-sitosterol

			Cassipourol	*β*-Sitosterol	STD
Anti-Inflammatory Activity	COX-1	1	10.34	6.78	6.11
		2	10.03	6.57	5.95
		3	9.86	6.46	6.06
		**Mean**	**10.08**	**6.61**	**6.04**
		**SD**	**0.25**	**0.16**	**0.08**
		**SE**	**0.14**	**0.09**	**0.05**
	COX-2	1	7.59	5.07	4.59
		2	7.40	4.95	4.50
		3	7.41	4.95	4.65
		**Mean**	**7.47**	**4.99**	**4.58**
		**SD**	**0.10**	**0.07**	**0.08**
		**SE**	**0.06**	**0.04**	**0.05**
	5-LOX	1	12.11	7.79	7.56
		2	12.12	7.84	7.57
		3	11.74	7.91	7.34
		**Mean**	**11.99**	**7.85**	**7.49**
		**SD**	**0.21**	**0.06**	**0.13**
		**SE**	**0.12**	**0.04**	**0.07**

Indomethacin and zileuton are reference standards (STD) for COXs and LOX, respectively.

Suppl. Table S3

Data of the cytotoxic activity of cassipourol and *β*-sitosterol against human colon cancer (Caco-2) cell line

**Cassipourol**						
**Conc.** (μg/mL)	**0.00**	**31.13**	**62.50**	**125.00**	**250.00**	**500.00**
Mean OD	0.37	0.31	0.27	0.20	0.12	0.08
Viability %	100.00	96.91	81.91	67.61	51.59	22.42
**IC_{50} (μg/mL)**	**222.27 ± 35.40**					
***β*-Sitosterol**						
**Conc.** (μg/mL)	**0.00**	**31.13**	**62.50**	**125.00**	**250.00**	**500.00**
Mean OD	0.36	0.31	0.27	0.19	0.12	0.08
Viability %	100.00	93.42	73.52	65.17	49.73	21.61
**IC_{50} (μg/mL)**	**177.73 ± 25.89**					
**Indomethacin**						
**Conc.** (μg/mL)	**0.00**	**6.25**	**12.50**	**25.00**	**50.00**	**100.00**
Mean OD	0.34	0.27	0.21	0.14	0.11	0.07
Viability %	100.00	92.28	81.32	64.73	35.45	15.38
**IC_{50} (μg/mL)**	**35.25 ± 7.59**					

Indomethacin is a reference standard.

Suppl. Table S4

Data of the cytotoxic activity of cassipourol and *β*-sitosterol against human lung cancer (A549) cell line

Cassipourol						
**Conc.** (μg/mL)	**0.00**	**6.25**	**12.50**	**25.00**	**50.00**	**100.00**
Mean OD	0.37	0.31	0.28	0.19	0.11	0.07
Viability %	100.00	97.24	81.74	67.42	51.11	21.41
**IC_{50} (μg/mL)**	**89.84 ± 5.65**					
***β*-Sitosterol**						
**Conc.** (μg/mL)	**0.00**	**6.25**	**12.50**	**25.00**	**50.00**	**100.00**
Mean OD	0.35	0.29	0.27	0.18	0.11	0.07
Viability %	100.00	92.08	79.18	63.84	48.40	20.28
**IC_{50} (μg/mL)**	**53.94 ± 4.39**					
**Indomethacin**						
**Conc.** (μg/mL)	**0.00**	**6.25**	**12.50**	**25.00**	**50.00**	**100.00**
Mean OD	0.31	0.21	0.17	0.12	0.10	0.06
Viability %	100.00	83.25	61.19	29.25	18.97	7.60
**IC_{50} (μg/mL)**	**25.73 ± 2.88**					

Indomethacin is a reference standard.

Suppl. Table S5
Common targets between inflammation, cassipourol and β-sitosterol, ranked according to the degree of involvement as revealed by compound-target-disease network

Protein code	Name	Degree
P17252	Protein kinase C alpha (PRKCA)	3
Q99685	Monoglyceride lipase (MGLL)	3
P14416	Dopamine receptor D2 (DRD2)	3
Q16665	Hypoxia inducible factor 1 subunit alpha (HIF1A)	3
Q07869	Peroxisome proliferator activated receptor alpha (PPARA)	3
P80365	Hydroxysteroid 11-beta dehydrogenase 2 (HSD11B2)	3
P06737	Glycogen phosphorylase L (PYGL)	3
P49682	C-X-C motif chemokine receptor 3 (CXCR3)	3
P24723	Protein kinase C eta (PRKCH)	3
Q00987	MDM2 proto-oncogene (MDM2)	3
O60725	Isoprenylcysteine carboxyl methyltransferase (ICMT)	3
Q05193	Dynamin 1(DNM1)	3
P06746	DNA polymerase beta (POLB)	3
P16662	UDP glucuronosyltransferase family 2 member B7 (UGT2B7)	3
P35228	Nitric oxide synthase 2 (NOS2)	3
Q14534	Squalene epoxidase (SQLE)	3
Q9UBM7	7-Dehydrocholesterol reductase (DHCR7)	3
P24557	Thromboxane A synthase 1 (TBXAS1)	3
P55055	nuclear receptor subfamily 1 group H member 2 (NR1H2)	3
P06276	Butyrylcholinesterase (BCHE)	3
P23975	Solute carrier family 6 member 2 (SLC6A2)	3
P33261	Cytochrome P450 family 2 subfamily C member 19 (CYP2C19)	3
P18031	Protein tyrosine phosphatase non-receptor type 1(PTPN1)	3
P10275	Androgen receptor (AR)	3
Q12772	Sterol regulatory element binding transcription factor 2 (SREBF2)	3
P04278	Sex hormone binding globulin (SHBG)	3
P51449	RAR related orphan receptor C (RORC)	3
Q13133	Nuclear receptor subfamily 1 group H member 3 (NR1H3)	3
Q9UHC9	NPC1 like intracellular cholesterol transporter 1 (NPC1L1)	3
P06493	Cyclin dependent kinase 1 (CDK1)	2
P11229	Cholinergic receptor muscarinic 1 (CHRM1)	2
P09917	Arachidonate 5-lipoxygenase (ALOX5)	2
P08069	Insulin like growth factor 1 receptor (IGF1R)	2
Q13464	Rho associated coiled-coil containing protein kinase 1 (ROCK1)	2
O75116	Rho associated coiled-coil containing protein kinase 2 (ROCK2)	2
P35968	Kinase insert domain receptor (KDR)	2
P53985	Solute carrier family 16 member 1 (SLC16A1)	2
P22748	Carbonic anhydrase 4 (CA4)	2

P00915	Carbonic anhydrase 1 (CA1)	2
P43166	Carbonic anhydrase 7 (CA7)	2
P00918	Carbonic anhydrase 2 (CA2)	2
P05129	Protein kinase C gamma (PRKCG)	2
Q05655	Protein kinase C delta (PRKCD)	2
P48730	Casein kinase 1 delta (CSNK1D)	2
P21730	Complement C5a receptor 1 (C5AR1)	2
Q05469	Lipase E, hormone sensitive type (LIPE)	2
O14965	Aurora kinase A (AURKA)	2
P07711	Cathepsin L (CTSL)	2
O15530	3-Phosphoinositide dependent protein kinase 1 (PDPK1)	2
Q96GD4	Aurora kinase B (AURKB)	2
O75874	Isocitrate dehydrogenase (NADP(+)) 1 (IDH1)	2
P49356	CHURC1-FNTB readthrough (CHURC1-FNTB)	2
Q9UBN7	Histone deacetylase 6 (HDAC6)	2
P08842	Steroid sulfatase (STS)	2
Q07973	Cytochrome P450 family 24 subfamily A member 1 (CYP24A1)	2
O60674	Janus kinase 2 (JAK2)	2
O00141	Serum/glucocorticoid regulated kinase 1 (SGK1)	2
P11309	Pim-1 proto-oncogene, serine/threonine kinase (PIM1)	2
P18405	Steroid 5 alpha-reductase 1 (SRD5A1)	2
O95819	Mitogen-activated protein kinase kinase kinase kinase 4 (MAP4K4)	2
Q15759	Mitogen-activated protein kinase 11 (MAPK11)	2
P51686	C-C motif chemokine receptor 9 (CCR9)	2
P07333	Colony stimulating factor 1 receptor (CSF1R)	2
Q13093	Phospholipase A2 group VII (PLA2G7)	2
P42330	Aldo-keto reductase family 1 member C3 (AKR1C3)	2
Q9Y5Z0	Beta-secretase 2 (BACE2)	2
O15151	MDM4 regulator of p53 (MDM4)	2
Q15722	Leukotriene B4 receptor (LTB4R)	2
O00763	Acetyl-CoA carboxylase beta (ACACB)	2
P14555	Phospholipase A2 group IIA (PLA2G2A)	2
P07099	Epoxide hydrolase 1 (EPHX1)	2
P50579	Methionyl aminopeptidase 2 (METAP2)	2
Q16875	6-Phosphofructo-2-kinase/fructose-2,6-biphosphatase 3 (PFKFB3)	2
P11802	Cyclin dependent kinase 4 (CDK4)	2
P21731	Thromboxane A2 receptor (TBXA2R)	2
P00533	Epidermal growth factor receptor (EGFR)	2
O15111	Component of inhibitor of nuclear factor kappa B kinase complex (CHUK)	2
P04062	Glucosylceramidase beta 1 (GBA1)	2
O14920	Inhibitor of nuclear factor kappa B kinase subunit beta (IKBKB)	2
Q15059	Bromodomain containing 3 (BRD3)	2

P25440	Bromodomain containing 2 (BRD2)	2
O60885	Bromodomain containing 4 (BRD4)	2
P20292	Arachidonate 5-lipoxygenase activating protein (ALOX5AP)	2
P28329	Choline O-acetyltransferase (CHAT)	2
Q9NRA0	Sphingosine kinase 2 (SPHK2)	2
O75582	Ribosomal protein S6 kinase A5 (RPS6KA5)	2
P49286	Melatonin receptor 1B (MTNR1B)	2
P48039	Melatonin receptor 1A (MTNR1A)	2
P49841	Glycogen synthase kinase 3 beta (GSK3B)	2
P25116	Coagulation factor II thrombin receptor (F2R)	2
P34972	Cannabinoid receptor 2 (CNR2)	2
P02753	Retinol binding protein 4 (RBP4)	2
Q14416	Glutamate metabotropic receptor 2 (GRM2)	2
P06401	Progesterone receptor (PGR)	2
P34913	Epoxide hydrolase 2 (EPHX2)	2
P21554	Cannabinoid receptor 1 (CNR1)	2
P30304	Cell division cycle 25A (CDC25A)	2
P05305	Endothelin 1 (EDN1)	2
P01137	Transforming growth factor beta 1	2
Q14790	Caspase 8 (CASP8)	2
P54646	Protein kinase AMP-activated catalytic subunit alpha 2 (PRKAA2)	2
P11137	Microtubule associated protein 2 (MAP2)	2
P42574	Caspase 3 (CASP3)	2
Q07812	BCL2 associated X, apoptosis regulator (BAX)	2
P10415	BCL2 apoptosis regulator (BCL2)	2
P55211	Caspase 9 (CASP9)	2
O75907	Diacylglycerol O-acyltransferase 1 (DGAT1)	2
P32246	C-C Motif chemokine receptor 1 (CCR1)	2
P31213	Steroid 5 alpha-reductase 2 (SRD5A2)	2
O60307	Microtubule associated serine/threonine kinase 3 (MAST3)	2
P45983	Mitogen-activated protein kinase 8 (MAPK8)	2
P08235	Nuclear receptor subfamily 3 group C member 2 (NR3C2)	2
P11387	DNA topoisomerase I (TOP1)	2
Q13258	Prostaglandin D2 receptor (PTGDR)	2
P43115	Prostaglandin E receptor 3 (PTGER3)	2
P43088	Prostaglandin F receptor (PTGFR)	2
P01375	Tumor necrosis factor (TNF)	2
P00742	Coagulation factor X (F10)	2
P00734	Coagulation factor II, thrombin (F2)	2
P05107	Integrin subunit beta 2 (ITGB2)	2
P11388	DNA topoisomerase II alpha (TOP2A)	2
P02774	GC vitamin D binding protein (GC)	2

O43613	Hypocretin receptor 1 (HCRTR1)	2
O43614	hypocretin receptor 2 (HCRTR2)	2
Q15465	Sonic hedgehog signaling molecule (SHH)	2
Q07343	Phosphodiesterase 4B (PDE4B)	2
P27815	Phosphodiesterase 4A (PDE4A)	2
P24666	Acid phosphatase 1(ACP1)	2
P04054	Phospholipase A2 group IB (PLA2G1B)	2
P21453	Sphingosine-1-phosphate receptor 1 (S1PR1)	2
Q99500	Sphingosine-1-phosphate receptor 3 (S1PR3)	2
O60218	Aldo-keto reductase family 1 member B10 (AKR1B10)	2
Q06124	Protein tyrosine phosphatase non-receptor type 11 (PTPN11)	2
P27361	Mitogen-activated protein kinase 3 (MAPK3)	2
P0DMS8	Adenosine A3 receptor (ADORA3)	2
Q01959	Solute carrier family 6 member 3 (SLC6A3)	2
P07148	Fatty acid binding protein 1 (FABP1)	2
Q01469	Fatty acid binding protein 5 (FABP5)	2
P05413	Fatty acid binding protein 3 (FABP3)	2
O14746	Telomerase reverse transcriptase (TERT)	2
P15090	Fatty acid binding protein 4 (FABP4)	2
Q15125	EBP cholestenol delta-isomerase (EBP)	2
P43119	Prostaglandin I2 receptor (PTGIR)	2
Q99835	Smoothened, frizzled class receptor (SMO)	2
P54707	ATPase H+/K+ transporting non-gastric alpha2 subunit (ATP12A)	2
P14902	Indoleamine 2,3-dioxygenase 1 (IDO1)	2
P35408	Prostaglandin E receptor 4 (PTGER4)	2
Q99720	Sigma non-opioid intracellular receptor 1 (SIGMAR1)	2
P48147	Prolyl endopeptidase (PREP)	2
P04150	Nuclear receptor subfamily 3 group C member 1 (NR3C1)	2
P37231	Peroxisome proliferator activated receptor gamma (PPARG)	2
P37268	Farnesyl-diphosphate farnesyltransferase 1 (FDFT1)	2
P17706	Protein tyrosine phosphatase non-receptor type 2 (PTPN2)	2
P29350	Protein tyrosine phosphatase non-receptor type 6 (PTPN6)	2
P28845	Hydroxysteroid 11-beta dehydrogenase 1 (HSD11B1)	2
P23415	Glycine receptor alpha 1 (GLRA1)	2
Q03181	Peroxisome proliferator activated receptor delta (PPARD)	2
O14684	Prostaglandin E synthase (PTGES)	2
P43116	Prostaglandin E receptor 2 (PTGER2)	2
P34995	Prostaglandin E receptor 1 (PTGER1)	2
P11473	Vitamin D receptor (VDR)	2
Q14994	Nuclear receptor subfamily 1 group I member 3 (NR1I3)	2
P08172	Cholinergic receptor muscarinic 2 (CHRM2)	2
P31645	Solute carrier family 6 member 4 (SLC6A4)	2

P11413	Glucose-6-phosphate dehydrogenase (G6PD)	2
P08185	Serpin family A member 6 (SERPINA6)	2
P22303	Acetylcholinesterase (Yt blood group) (ACHE)	2
Q92731	Estrogen receptor 2 (ESR2)	2
P03372	Estrogen receptor 1 (ESR1)	2
P35398	RAR related orphan receptor A (RORA)	2
Q16850	Cytochrome P450 family 51 subfamily A member 1 (CYP51A1)	2
P11511	Cytochrome P450 family 19 subfamily A member 1 (CYP19A1)	2
P05093	Cytochrome P450 family 17 subfamily A member 1 (CYP17A1)	2
P04035	3-Hydroxy-3-methylglutaryl-CoA reductase (HMGCR)	2
P49354	Farnesyltransferase, CAAX box, subunit alpha (FNTA)	1
P24385	Cyclin D1 (CCND1)	1
P20701	Integrin subunit alpha L (ITGAL)	1
P05362	Intercellular adhesion molecule 1 (ICAM1)	1

The UniProt codes were converted to their corresponding protein names using SynGo ID conversion tool (https://www.syngoportal.org/convert, accessed on 23 November 2024).

Suppl. Table S6
Common targets between inflammation, cassipourol and β-sitosterol, ranked according to the degree of involvement as revealed by PPI

Target	Degree
EGFR	30
TNF	25
MAPK3	24
HIF1A	24
BCL2	24
CASP3	21
ESR1	19
MDM2	18
CCND1	17
JAK2	15
AR	15
PPARG	13
CYP19A1	13
CASP9	12
CASP8	12
NR3C1	12
CDK1	12
MAPK8	12
IGF1R	12
PPARA	11
CDK4	11
PGR	11
GSK3B	11
DHCR7	10
SREBF2	10
PRKCA	10
TGFB1	9
AKR1C3	9
CYP17A1	8
ESR2	8
KDR	8
PTPN11	8
AURKA	8
ICAM1	8
FDFT1	8
LIPE	8
HMGCR	8
FABP4	7
HDAC6	7

BRD4	7
PTPN6	7
PTGES	7
SRD5A1	7
CYP51A1	7
PTPN1	6
SPHK2	6
SRD5A2	6
CYP24A1	6
TOP2A	6
MAPK11	6
CHUK	6
SQLE	6
EDN1	6
ITGB2	6
PRKCD	5
F2	5
PRKCG	5
HSD11B1	5
CDC25A	5
PDPK1	5
CCR1	5
ALOX5	5
UGT2B7	5
FABP5	5
CXCR3	5
IKBKB	5
HSD11B2	5
NR3C2	5
DRD2	5
SHBG	4
PRKAA2	4
NR1H3	4
STS	4
SIGMAR1	4
GC	4
BAX	4
PLA2G2A	4
CA2	4
CYP2C19	4
F2R	4
AURKB	4
SHH	4

ROCK1	4
TERT	4
PPARD	4
ITGAL	3
MGLL	3
CSF1R	3
CNR1	3
CSNK1D	3
FABP1	3
SLC6A3	3
CA1	3
BRD2	3
FNTA	3
SMO	3
PTGER4	3
IDH1	3
EBP	3
CHAT	3
PTPN2	3
CCR9	3
PLA2G1B	3
ALOX5AP	2
SERPINA6	2
NR1H2	2
CHRM2	2
RORA	2
PLA2G7	2
PTGER3	2
S1PR1	2
PTGER2	2
SGK1	2
FABP3	2
CHRM1	2
MDM4	2
G6PD	2
S1PR3	2
CA7	2
AKR1B10	2
TOP1	2
PFKFB3	2
PTGER1	2
ATP12A	2
RPS6KA5	2

ROCK2	2
FNTB	2
PTGFR	2
BCHE	2
BRD3	2
NPC1L1	2
DGAT1	2
EPHX1	2
GRM2	1
C5AR1	1
MAP2	1
PTGIR	1
F10	1
HCRTR2	1
HCRTR1	1
EPHX2	1
ACP1	1
CNR2	1
ACHE	1
RBP4	1
RORC	1
IDO1	1
SLC16A1	1
LTB4R	1
ACACB	1
SLC6A4	1
MTNR1A	1
NOS2	1
CTSL	1
CA4	1
TBXA2R	1
MTNR1B	1
PRKCH	0
SLC6A2	0
ICMT	0
PREP	0
ADORA3	0
TBXAS1	0
MAP4K4	0
POLB	0
NR1I3	0
PTGDR	0
DNM1	0

METAP2	0
GBA	0
BACE2	0
MAST3	0
GLRA1	0
PIM1	0
PDE4B	0
PYGL	0
PDE4A	0
